# Physical and chemical properties of aloe-vera coated guava *(Psidium guajava)* fruit during refrigerated storage

**DOI:** 10.1371/journal.pone.0293553

**Published:** 2023-11-01

**Authors:** Debashis Kumar Dutta Roy, Md. Asaduzzaman, Tanny Saha, Mst. Nazma Khatun

**Affiliations:** 1 Department of Food Processing and Preservation, Hajee Mohammad Danesh Science and Technology University, Dinajpur, Bangladesh; 2 Department of Food Engineering and Technology, State university of Bangladesh, Dhaka, Bangladesh; 3 Department of Food Technology, Chapainawabganj Polytechnic Institute, Chapainawabganj, Bangladesh; University of Jeddah, SAUDI ARABIA

## Abstract

Guavas (Psidium guajava) are regarded as one of the most perishable commodities, primarily owing to their climacteric characteristics and heightened metabolic processes, resulting in a faster rate of softening. Edible coating is a natural ingredient that is employed as an alternative to extend the shelf life of fruits while also providing bioactive and functional compounds. Aloe vera gel is predominantly used for this purpose due to its widespread availability. Various concentrations of aloe vera-based coating formulation (25%, 50%, 75%, and 100%) were applied on fresh whole guava by dipping method. The guava was stored at a refrigerated condition (4°C) and weight loss, color, firmness, vitamin C, total phenol, and pH change were observed in this research. A significant effect of aloe vera coating was found over the storage period. Aloe vera treatment lowered the weight loss, and retarded the texture and color compared to the control sample throughout the 28 days of storage. Vitamin C and total phenol content remined high at 141.4 mg/100g and 219.6 mg GAE/100g respectively in a 100% aloe vera coated sample after 28 days of storage compared to the control. Among 25%, 50%, 75%, and 100% aloe vera coated sample, 100% aloe vera was found to be the best coating material to prevent physical changes in fresh guava.

## Introduction

Guava (*Psidium guajava*) is one of the most common tropical fruits in Bangladesh. Guava is popular for its high content of antioxidants such as vitamin C, 260 mg per 100 gm [1, 2]. It is also rich in antioxidant and bioactive compounds which can retard aging by reducing oxidative damage of lipids, protein and nucleic acid [2]. According to Hossen, 2012, the annual production of guava is about 145,000 m tons in an area of about 10,000 hectors per year on an average of which 30–40% is wasted due to several causes in Bangladesh due to highly perishable and low shelf-life ranges from 3 to 10 days at room temperature [3]. Without any treatment guava is spoiled very easily because of its high respiration rates, mechanical damage, and microbial decomposition. Different method including ionizing radiation, preservatives, and controlled atmosphere are used for the preservation of guava [4–6].

Additionally application of edible films and coating can increase the shelf life of guava by reducing moisture content and gas loss as well as improve the quality, safety, transportation, storage, and display of a wide range of fresh and processed fruits [7–9]. However, edible films and coatings also may prevent moisture loss and contamination of fruits and vegetables [9]. They can act as moisture and oxygen barrier during processing, handling, transportation [8]. During storage, it helps to retard decomposition and enhance the safety of guava by improving antimicrobial functions [10]. In recent studies; the edible coating formulation is used as a new improved technique that acts as a packaging material. However, it has more functional properties than thermoplastic materials [11].

Different types of edible coating are used and aloe vera is one of them [7, 12]. Aloe vera gel is a polysaccharide-based edible coating material, which has a commercial application in the processing of fruits [13]. It has therapeutic values which can an innovative and interesting means for commercial application. *Aloe* v*era* has been used for centuries for its medicinal and therapeutic, antioxidant, and anti-microbial properties [14–17]. Various types of coating conditions and different coating materials can enhance the shelf life of different fruits [16]. The annual production of aloe vera exceeds 1000 metric tonnes, primarily utilized in Ayurvedic, Unani, and herbal treatments [18].

Though a large quantity of guava is wasted during post-harvest handling, coating of guava can reduce this type of loss, so in this study, we will evaluate the concentration of aloe vera coating for guava as well as analyze the effect of various concentrations of aloe vera on physical and chemical properties of fresh and coated guava over the storage period.

## Materials and methods

### Experimental site

The study was conducted in the laboratory of Food Engineering & Technology and Food Processing & Preservation department under the Faculty of Engineering of Hajee Mohammad Danesh Science and Technology University, Dinajpur. The study was conducted in the chemistry laboratory of this university. Samples and chemical collection Fresh guava and Aloe vera leaves were bought from local market, Dinajpur. Guava fruits of uniform size and shape were selected. All analytical grade chemicals such as Glycerol, methanol, Glacial acetic acid, Meta phosphoric acid were supplied by the food processing laboratory, Hajee Mohammad Danesh Science and Technology University, Dinajpur.

### Preparation of aloe vera gel

Aloe vera leaves were washed with water and skins were peeled. The gels were separated, collected and ground in a blender. Then aloe vera pulp was filtered to remove the fibres [19].

### Preparation of coating materials

Edible coating material was made by adding 1% glycerol (used as plasticizing agent), 5% Corn starch (used as a thickening agent), 5% ethanol (used for fast drying of coating), and 1% acetic acid with Aloe vera gel and stirred evenly for 20 minutes. Then the aloe vera gel solution was heated at a temperature of 70°C for 45 minutes to pasteurize it and cooled to room temperature and stored in the refrigeration temperature.

### Guava coating process

Fresh guavas were then dipped into with different concentrations of aloe vera (T_0_ = No Aloe Vera, T_1_ = 25%, T_2_ = 50%, T_3_ = 75%, and T_4_ = 100%) for 2 minutes and then dried naturally for 30 minutes.

After drying the coated guavas were packed using LDPE and stored refrigerated at 4°C for 30 days.

### Physical properties

#### Determination of pH

The pH of the selected samples was determined by the conventional procedure of a pH meter.

#### Weight loss

Sample weight loss was determined by comparing the weights of coated guava after storage with initial weights and expressing the results as a percentage (Chen *et al*., 2007). The results are calculated with the following equation where W_1_ was the Initial weight and W_2_was the Final weight.


$$Weight\;loss=\frac{W_{1}{-W}_{2}}{W_{1}}\times 100$$

#### Firmness

The firmness of Guava was determined by using a penetrometer. The firmness test was done by penetrating the stainless steel probe 3.5 mm in diameter. The probe was penetrating up to three different locations in each sample. The data was recorded as a force expressed as a unit of Newtons (N).

#### Color

The color property of guava was determined with a Colorimeter Minolta CM-2500d (Konica Minolta Optics, Inc. Japan). Color attributes were recorded as L* (lightness), a* (redness), and b* (yellowness). Here the change in lightness and Hue angle is calculated. The equation used for hue is as follows where h = Hue angle, b = yellowness, and a = redness

$$\text{h}={\text{tan}}^{-1}\;(\text{b}/\text{a}).$$

### Chemical properties

#### Proximate composition

Moisture, crude protein, fat, and ash content of flours were determined by official methods (AOAC, 1998) [20].

#### Vitamin C

The vitamin C content of guava was measured using the AOAC method (2,6-Dichloroindophenol titrimetric method, 2006).

#### Total phenol

Total phenol was determined according to the method described by Lin &Tang, 2007., with slight modifications [21]. The absorbance was taken at 760 nm. The phenol content was determined using the Gallic acid standard curve and was expressed as ppm.

Total phenol content was calculated by the following formula:

$$\%\;Phenol=X\;(ppm)\times \frac{Total\;volume\;made\;up}{Weight\;of\;sample(mg)}\times 100$$

### Statistical analysis

Each experiment included three replications. Data were analyzed using statistical software (R-version 3.2.2.). A multifactorial analysis of variance was carried out. Individual effects of the factors have been calculated at a particular point in time during the study. Differences were considered to be significant at P<0.05.

## Results and discussion

### Physical properties

#### pH

**Fig 1:**
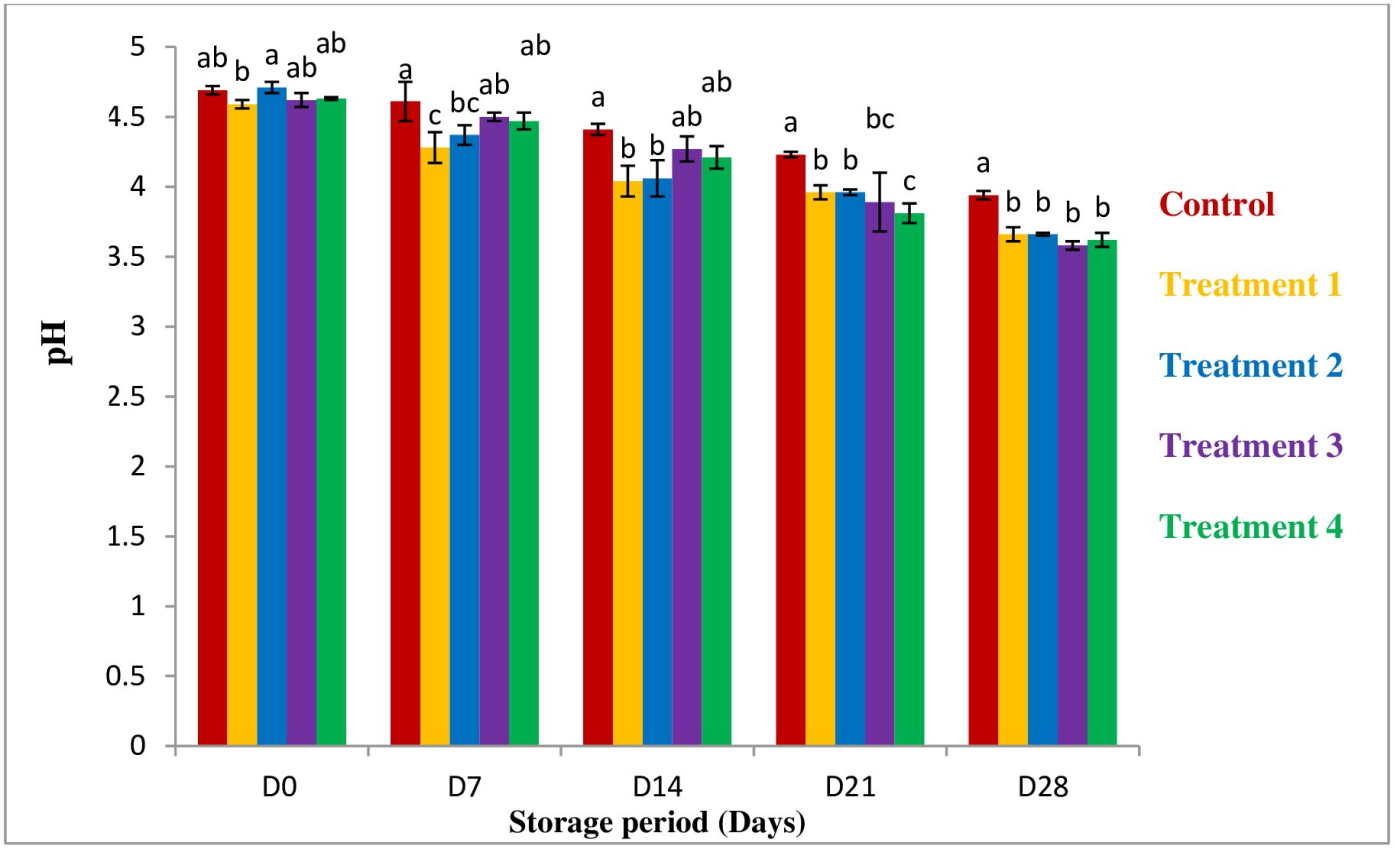
pH values in different storage period. *All values are express as mean ± SD. *Mean followed by different superscript letters in each raw are significantly different (p<0.05).

The pH content of guava decreased gradually with the increase of storage time (Fig 1). The 100% aloe vera coated sample (Treatment 04) shows the lowest pH 3.62 at day 28 compared with the others with significantly different (P>0.05). Normally the lower pH is create difficult condition to grow microorganisms which means 100% aloe vera controls microbial growth of microbes [17]. According to Keditsu, this pH change due to presence of the organic acids and conversion of sugar into acid and metabolic processes [22].

#### Weight loss

**Fig 2:**
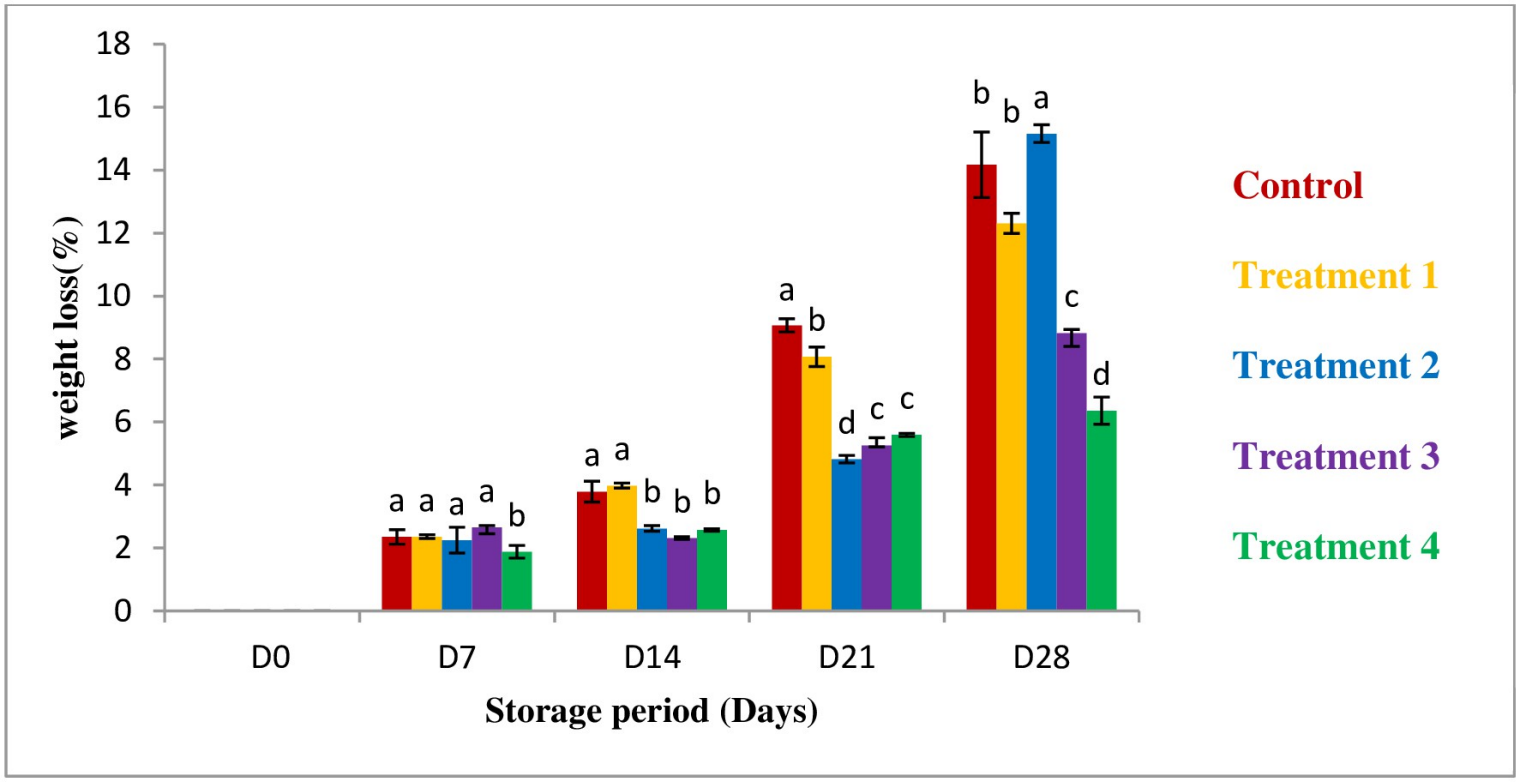
Effect of coating on weight loss. *All values are express as mean ± SD. *Mean followed by different superscript letters in each raw are significantly different (p<0.05).

The weight loss was measured up to 35 days, here the data is shown28 days (Fig 2). The change in weight loss was increased through the storage period. The highest weight loss was found in the control sample compared with the coated ones. The highest weight loss was 14.17% in control where the lowest value was 6.36% for 100% Aloe vera coated guava Though the weight loss is different is different coating percentages but 100% Aloe vera achieved the lowest weight loss in 28 days which ensures he weight loss was minimized by aloe vera coating. Similar observation proved by Dorria (2007) & Zuraidah (2015) [23, 24]. Normally, Weight loss of guava occurs due to dehydration, respiration, and physical cell disruption [25].

#### Firmness

**Fig 3:**
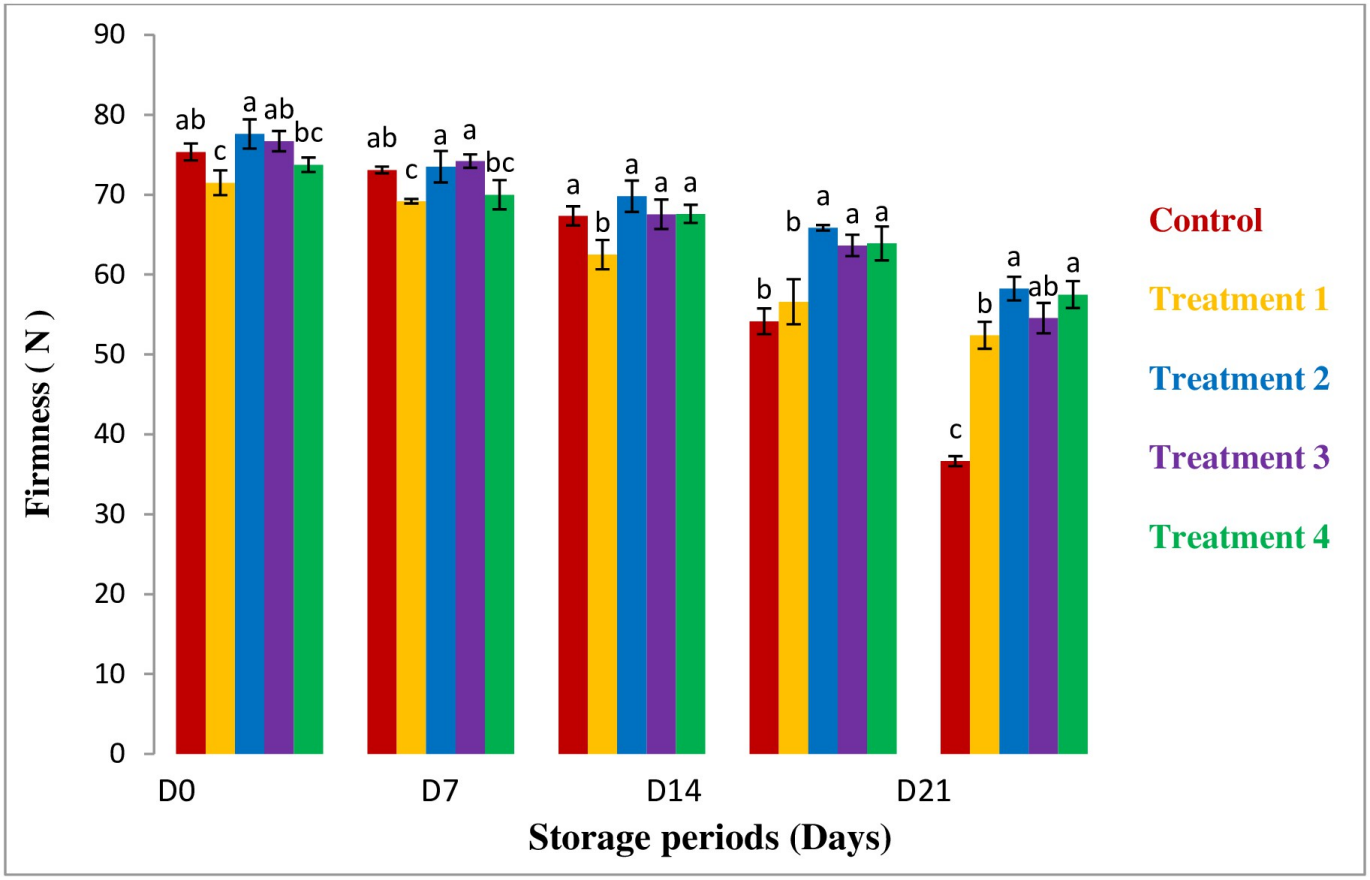
Changes in firmness in the coating samples. *All values are express as mean ± SD. *Mean followed by different superscript letters in each raw are significantly different (p<0.05).

Weight loss can affect the firmness of guava. The softness of guava is also affected by ethylene activity which is highest at room temperature within 4 days at room temperature [26, 27]. Fig 3 represents that the firmness is gradually lowered by increasing the storage period. In the case of coated samples the firmness is higher than the uncoated ones and 100% aloe vera coating restricts the firmness loss during the 28 days of storage period. Martinez–Romero *et al*., (2006) also found that aloe vera gel has the power to lower weight loss which also influences the retarding of firmness [28]. The possible reason may be the use of a higher concentration of aloe vera in this study. The higher concentration of aloe vera retards the firm loss and improved quality [24, 28].

#### The color attribute of guava

**Fig 4:**
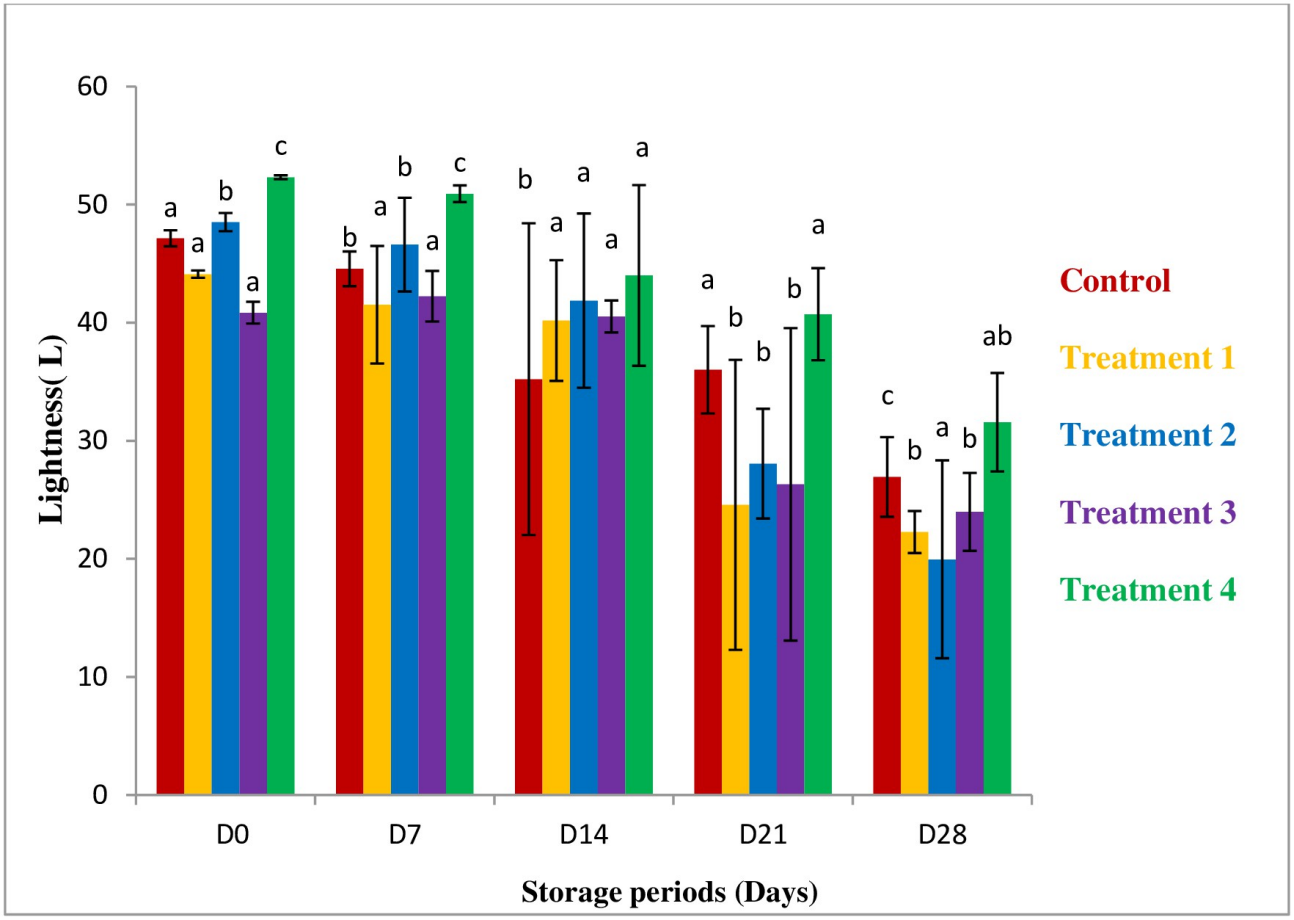
Effect of coating on lightness. *All values are express as mean ± SD. *Mean followed by different superscript letters in each raw are significantly different (p<0.05).

The change in lightness, redness, and yellowness is shown in Fig 4 and Table 1. The value showed negative in the case of “a” because guava is generally green in color. When storage time was increased lightness of guava decreased (highest 47.15 to 26.94 in the controlled and 52.32 to 31.57 in the 100% aloe vera coated sample). But the coated sample has higher lightness than the uncoated sample. On day 28, the 100% aloe vera-coated sample had higher value than others. One of the possible reasons for the change in lightness is the coating material. It acts as a modified atmosphere packaging material which delays the degradation of color [29]. According to Baldwin *et al*.,1995 the edible coating was also demonstrated to delays color change, and weight loss in tropical fruit and improves appearance [7].

**Table 1:** Comparison of different color parameter (a, b and Hue values) for different samples of the product.

Treatment	Storage periods (Days)				
	D_0_	D_7_	D_14_	D_21_	D_28_
**a value**					
T_0_	-8.86±2.05^a^	-25.9±14.1^a^	-31.79±29.4^c^	-27.12±7.70^b^	-15.55±5.44^b^
T_1_	-6.64±2.05^a^	-15.46±0.5^ab^	-21.45±7.61^a^	-12.03±5.49^a^	-8.77±6.09^b^
T_2_	-8.98±2.14^a^	-19.92±2.1^ab^	-29.81±16.8^ab^	-13.16±3.23^a^	-13.99±0.65^a^
T_3_	-5.97±1.97^a^	-21.56±8.64^a^	-13.5±11.5^ab^	-20.1±0.79^ab^	-10.60±0.37^b^
T_4_	-8.08±2.12^a^	-8.30±0.21^a^	-25.14±9.5^b^	-32.11±5.25^b^	-16.1±0.81^b^
**b value**					
T_0_	32.65±0.40^a^	10.16±0.76^b^	14.85±1.15^ab^	14.30±2.99^b^	13.63±4.6^b^
T_1_	28.34±0.28^b^	15.98±8.72^b^	26.33±4.56^a^	24.96±9.34^a^	18.44±2.52^a^
T_2_	31.55±0.40^a^	17.26±2.25^c^	15.3±4.39^ab^	15.805±0.86^b^	17.94±7.21^a^
T_3_	27.91±1.19^b^	32.46±1.26^a^	24.12±12.2^a^	20.04±6.39^a^	17.33±0.62^a^
T_4_	31.64±0.27^a^	19.45±0.17^c^	17.06±1.13^b^	18.36±4.54^a^	17.74±0.94^a^
**Hue angle (h** ^**0**^ **)**					
T_0_	-74.85±3.14^a^	-23.87±10.2^b^	-34.97±28.3^a^	-28.83±11.7^a^	-51.72±5.02^c^
T_1_	-74.64±0.71^a^	-43.73±15.1^bc^	-51.05±14.6^ab^	-62.255±18^b^	-64.72±18.1^b^
T_2_	-64.1±10.7^ab^	-40.9±0.74^bc^	-52.1±41.1^b^	-49.08±7.5^ab^	-50.66±10.3^a^
T_3_	-78.02±3.40^b^	-57.12±9.61^c^	-58±32.0^ab^	-44.13±8.1^ab^	-58.54±0.03^ab^
T_4_	-75.68±3.73^a^	-66.88±0.73^a^	-36.19±8.72^a^	-29.56±2.11^a^	-47.77±0.08^a^

*All values are express as mean ± SD.

*Mean followed by different superscript letters in each raw are significantly different (p<0.05).

*T_0_ = Control, T_1_ = 25% Aloe vera, T_2_ = 50% Aloe vera,T_3_ = 75% Aloe vera, T_4_ = 100% Aloe vera.

In the case of greenness, a lowering effect throughout the storage period was observed. The green color changed from light yellow to dark yellow. The controlled sample changed its color earliest because of its high respiration rate compared to the other coated samples. Previous studies also showed the same color changes over the period [30, 31].

With the change of time, the hue angles were increased (Table 1). This indicates the changing of green to yellow color. The reduction of greenness (-8.08 to -16.01) is nearly similar to Xing et al., 2011, who used a polysaccharide-based chitosan coating [32].

### Chemical properties

#### Proximate analyses

The chemical composition of guava and aloe vera is shown in Table 2. In the case of guava, the results showed 83.73% moisture, 12.6% carbohydrates, 1.7% ash, and 1.3% protein, which were similar to those reported by USDA (1982) [33]. They found 80.61% moisture, 0.7% ash, 1.28% acidity, 11% carbohydrates, and 19% TSS. The variation in the results may be due to different species, environmental conditions, and horticultural practices in guava production [34].

**Table 2:** Proximate composition of guava and aloe vera.

Sample	Composition (%)						
	Moisture	Protein	Fat	Carbohydrate	Ash	TSS	pH
Guava	83.73±0.73	1.3±0.04	-	12.6±0.24	1.7±0.16	18.6±0.45	4.63±0.21
Aloe vera	97.4±0.52	-	-	1.7±0.07	1.3±0.08	1.2±0.36	4.76±0.16

*All values are express as mean ± SD.

*Mean followed by different superscript letters in each raw are significantly different (p<0.05).

On the other hand, in the case of aloe vera, the results showed 97.4% moisture, 1.3% ash, and 1.7% carbohydrates. Pierce (1983) and Rowe (1941) also found 98.5% moisture and 0.3% carbohydrates, which were nearly similar [35, 36].

#### Vitamin C

**Fig 5:**
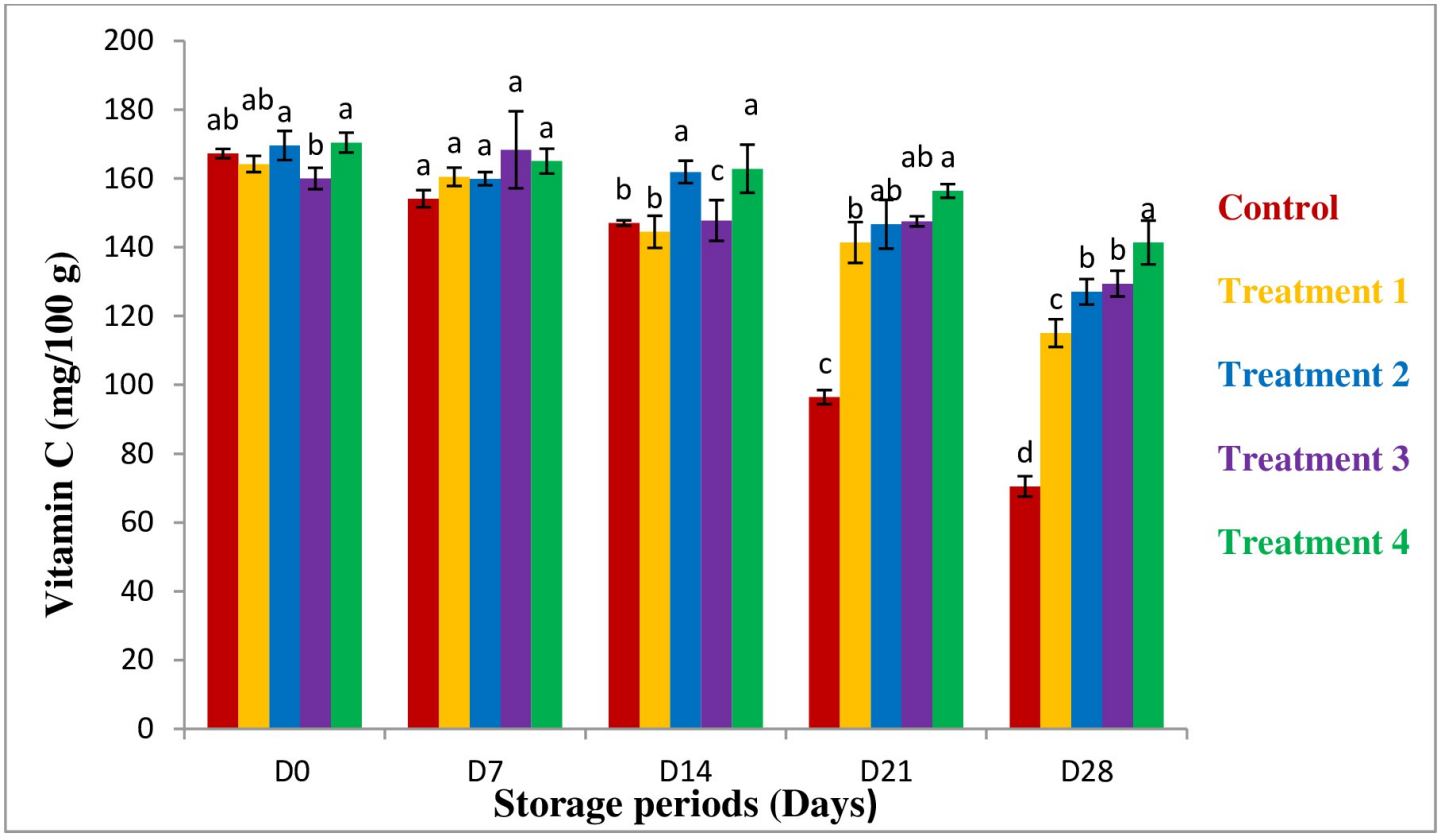
Vitamin C content of the coating samples. *All values are express as mean ± SD. *Mean followed by different superscript letters in each raw are significantly different (p<0.05).

Vitamin C is an important factor for guava. The change in vitamin C of guava is shown in Fig 5. In this study, the result shows vitamin C content of raw guava was 167.25 mg/100g which was similar to Vila *et al*., 2007 [37].

During the storage period, the vitamin C content of guava is decreased in all samples. The loss of vitamin C in the control sample is 96.75mg/100g and in the coated sample were 49.46, 42.55, 30.55, and 29.05mg/100g respectively. The result shows that vitamin C loss was higher in the fresh sample than in the coated sample. Vitamin C loss may occur because of phenol oxidase and ascorbic acid oxidase [38]. Also, the presence of light, heat, and oxygen can reduce the vitamin C content [39]. On the other hand modified packaging (aloe vera coating) creates an aerobic condition on the guava surface which influenced the lower oxidation rate in the guava surface [40].

This study concluded that the loss of vitamin C in the aloe vera coated sample is lower than the control. The higher concentration of aloe vera has the higher the efficiency of preservation. In this study, 100% coating concentration shows a higher result which is similar to Serrano *et al*., 2006 [41].

#### Total phenol content

Total phenol is also an important factor for the maturity index. Table 3 shows the changes in total phenol throughout the storage periods. The result showed that the total phenol content of guava in the fresh sample was 265.3–271.1 mg GAE per 100 g. This result is similar to Thaipong *et al*., 2006, and Hagen *et al*., 2007 who reported that it could be 170–344 mg GAE per 100g [42, 43].

**Table 3:** Effect of coating on total phenol content of guava (mg GAE per 100 g).

Treatment	Storage periods (Day)				
	D_0_	D_7_	D_14_	D_21_	D_28_
T_0_	265.3±0.56^ab^	255.6±4.66^d^	193.6±1.83^d^	172.7±5.79^c^	133.85±6.29^d^
T_1_	256.45±2.61^c^	242.7±4.10^b^	211.65±1.34^c^	196.75±3.60^b^	173.15±5.30^c^
T_2_	263.25±2.62^b^	251.85±4.59^ab^	230.55±2.61^b^	239.25±9.12^a^	190.35±3.46^b^
T_3_	249.2±3.39^d^	242.05±4.45^b^	225±5.09^b^	212.65±8.83^b^	194.4±2.82^b^
T_4_	271.1±1.83^a^	256.95±2.33^d^	245.3±1.97^a^	230.4±3.26^a^	219.6±2.40^a^

*All values are express as mean ± SD.

*Mean followed by different superscript letters in each raw are significantly different (p<0.05).

*T_0_ = Control, T_1_ = 25% Aloe vera, T_2_ = 50% Aloe vera,T_3_ = 75% Aloe vera, T_4_ = 100% Aloe vera.

During the storage periods, total phenol content was reduced to between 265.3 and133.85 for control and between 271.1 and219.6 mg GAE per 100g for the 100% aloe vera coated sample. The loss in the control sample was 131.45 and in the coated sample were 83.3, 72.9, 54.8, and 51.5 mg GAE per 100g respectively. The change may be due to many factors such as variety, species, ripening, and environmental conditions [44]. According to Taylor (1993), the lowering in total phenol content can reduce the astringency during the storage period [45]. More monomeric components can convert to polymeric components; these also cause the reduction of total phenol [46, 47]. This study shows that the loss of total phenol is reduced by the application of aloe vera coating which is also proven by Serrano *et al*., 2006 [41].

#### Storage studies of guava

Organoleptic properties of all the samples of guava were observed during storage periods at refrigeration temperature (4°C), based on color, flavor, texture, and visual fungal growth. This was recorded at every 7 days intervals up to 35 days. The data are shown in Table 4. In the case of the control sample, there was no change in the properties for up to 7 days. On day 14 the color started to change from dark green to green and slight fungal growth was seen. On day 21 it was changed its all property and organoleptically unaccepted for consume because excessive fungal growth.

**Table 4:** Sensory studies of coated guava.

Storage period	Sample	Color	Flavor	Texture	Visual fungal growth	Remarks
Day 0	T_0_	Dark Green	Good	Hard	No growth	Good
	T_1_	Dark Green	Good	Hard	No growth	Good
	T_2_	Dark Green	Good	Hard	No growth	Good
	T_3_	Dark Green	Good	Hard	No growth	Good
	T_4_	Dark Green	Good	Hard	No growth	Good
Day 7	T_0_	Dark Green	Good	Hard	No growth	Good
	T_1_	Dark Green	Good	Hard	No growth	Good
	T_2_	Dark Green	Good	Hard	No growth	Good
	T_3_	Dark Green	Good	Hard	No growth	Good
	T_4_	Dark Green	Good	Hard	No growth	Good
Day 14	T_0_	Green	Good	Semi Soft	Slightly growth	Good
	T_1_	Dark Green	Good	Semi Soft	No growth	Good
	T_2_	Dark Green	Good	Semi Soft	No growth	Good
	T_3_	Dark Green	Good	Semi Soft	No growth	Good
	T_4_	Dark Green	Good	Semi Soft	No growth	Good
Day 21	T_0_	Slight yellowish	Off flavor	Soft	Highly growth	Spoiled
	T_1_	Green	Good	Semi Soft	No growth	Good
	T_2_	Green	Good	Semi Soft	No growth	Good
	T_3_	Green	Good	Semi Soft	No growth	Good
	T_4_	Green	Good	Semi Soft	No growth	Good
Day 28	T_0_	Fully yellowish	Off flavor	No texture	Excessive growth	Spoiled
	T_1_	Slight yellowish	Off flavor	Soft	Excessive growth	Spoiled
	T_2_	Slight yellowish	Off flavor	Soft	Excessive growth	Slightly Spoiled
	T_3_	Slight yellowish	Off flavor	Semi Soft	Medium growth	Slightly spoiled
	T_4_	Slight yellowish	Off flavor	Semi Soft	Medium growth	Slightly spoiled
Day 35	T_0_	Black	Off flavor	No texture	Excessive growth	Spoiled
	T_1_	Full yellow	Off flavor	No texture	Excessive growth	Spoiled
	T_2_	Full yellow	Off flavor	Semi Soft	Excessive growth	Spoiled
	T_3_	Full yellow	Off flavor	Semi Soft	Medium growth	Spoiled
	T_4_	Full yellow	Off flavor	Semi Soft	Medium growth	Spoiled

*T_0_ = Control, T_1_ = 25% Aloe vera, T_2_ = 50% Aloe vera,T_3_ = 75% Aloe vera, T_4_ = 100% Aloe vera.

In the case of the coated sample, there was no change up to 21 days except for a little of softness. At day 28, 25% and 50% aloe vera coated samples were unaccepted because they changed their properties. On the other hand, 75% and 100% coated samples had better quality than the other samples and were organoleptically acceptable for up to 28 days. But all samples were spoiled on day 35. Kendall and Sofos (2007) found that if the sample is processed properly and stored in a cool dry place it will retain good quality for up to several months [48].

## Conclusion

This study was conducted to introduce a novel processing technique, edible coating by aloe vera gel on guava with the objectives to document the physico-chemical properties and find the suitable concentration of coating during storage periods. The analyzed proximate composition of guava and aloe vera gel was 83.73% moisture, 1.3% protein, 12.6% carbohydrate, 1.7% ash, and 97.4% moisture, 1.2% ash, and 0.7% carbohydrate respectively. The weight loss and soft texture were found to be lower in the coated samples. The vitamin C content of guava was higher (141.4mg) in the coated samples than in the fresh guava (70.5mg) on the 28^th^ day of the storage period. The color change (yellowness) was also lower in the coated sample and had a better appearance and acceptance. The total phenol content loss was also controlled by the application of coating material. Therefore it is concluded that the application of aloe vera coating can increase the shelf life of guava. Also, the higher concentration of coating material has a higher quality. Among all the treatments 100% aloe vera coating was best. As it increased the shelf life and lowered the loss of nutritional and quality parameter, it can be used as a natural preservation technique.

## Supporting information

S1 File (DOCX)

S2 File (DOCX)

S1 Fig (JPG)
