# Supplementary material for: Physical and chemical properties of aloe-vera coated guava (Psidium guajava) fruit during refrigerated storage
Source: PLoS One. 2023 Nov 1;18(11):e0293553. doi: 10.1371/journal.pone.0293553 (PMC10619840; doi:10.1371/journal.pone.0293553)
Supplement: S1 File — (DOCX) [file pone.0293553.s001.docx]

**Figure 1 Data Table: Effect of aloe-vera coating on pH at refrigerated condition**

| **Treatment** | **Storage period (Day)** | | | | |
| --- | --- | --- | --- | --- | --- |
|  | D_0_ | D_7_ | D_14_ | D_21_ | D_28_ |
| T_0_ | 4.72  4.67  4.69 | 4.6  4.62  4.61 | 4.38  4.44  4.47 | 4.21  4.25  4.19 | 3.92  3.96  3.97 |
| T_1_ | 4.57  4.62  4.60 | 4.2  4.36  4.28 | 3.96  4.12  4.16 | 3.92  4.0  3.96 | 3.7  3.62  3.66 |
| T_2_ | 4.68  4.75  4.71 | 4.32  4.42  4.37 | 4.16  3.94  4.06 | 3.94  3.98  3.92 | 3.66  3.67  3.61 |
| T_3_ | 4.58  4.66  4.63 | 4.48  4.52  4.57 | 4.34  4.21  4.27 | 3.91  3.87  3.89 | 3.56  3.61  3.58 |
| T_4_ | 4.64  4.62  4.63 | 4.43  4.52  4.49 | 4.27  4.15  4.21 | 3.76  3.86  3.81 | 3.58  3.66  3.62 |

**Figure 2 Data Table: Effect of aloe-vera coating on weight loss at refrigerated condition**

| **Treatment** | **Storage period (Day)** | | | | |
| --- | --- | --- | --- | --- | --- |
|  | D_0_ | D_7_ | D_14_ | D_21_ | D_28_ |
| T_0_ | 0 | 2.35  2.68  2.38 | 3.79  3.32  3.72 | 9.07  8.76  9.21 | 14.17  12.69  15.89 |
| T_1_ | 0 | 2.36  2.45  2.57 | 3.98  3.86  3.64 | 8.07  7.63  8.89 | 12.31  12.75  11.72 |
| T_2_ | 0 | 2.25  2.83  2.49 | 2.62  2.48  2.76 | 4.82  4.65  4.92 | 15.16  14.76  15.58 |
| T_3_ | 0 | 2.65  2.74  2.47 | 2.31  2.39  2.13 | 5.26  5.60  4.93 | 8.83  8.67  9.03 |
| T_4_ | 0 | 1.88  1.59  2.06 | 2.57  2.63  2.49 | 5.59  5.65  5.51 | 6.36  6.96  5.72 |

**Figure 3 Data Table: Effect of aloe-vera coating on firmness at refrigerated condition**

| **Treatment** | **Storage period (Day)** | | | | |
| --- | --- | --- | --- | --- | --- |
|  | D_0_ | D_7_ | D_14_ | D_21_ | D_28_ |
| T_0_ | 76.1  74.6  75.3 | 72.8  73.4  73.1 | 66.5  68.2  67.3 | 53  55.3  54.1 | 37.1  36.2  35.9 |
| T_1_ | 72.6  70.4  71.5 | 68.9  69.3  70.2 | 63.8  61.2  60.6 | 58.6  54.6  54.3 | 53.6  51.2  49.6 |
| T_2_ | 78.9  76.3  77.6 | 74.9  72.1  73.5 | 71.2  68.4  69.8 | 65.6  66.1  65.3 | 57.2  59.3  58.1 |
| T_3_ | 77.6  75.8  76.5 | 74.8  73.6  74.1 | 68.6  66.5  67.5 | 64.6  62.7  63.2 | 55.9  53.2  54.1 |
| T_4_ | 74.4  73.1  73.8 | 71.3  68.7  69.6 | 68.4  66.8  66.7 | 65.4  62.4  62.9 | 56.3  58.7  57.2 |

**Figure 4 Data Table: Effect of aloe-vera coating on lightness at refrigerated condition**

| **Treatment** | **Storage period (Day)** | | | | |
| --- | --- | --- | --- | --- | --- |
| L value | D_0_ | D_7_ | D_14_ | D_21_ | D_28_ |
| T_0_ | 47.64  46.67  47.15 | 45.61  43.52  44.56 | 44.56  25.89  35.22 | 36.01  41.25  38.63 | 24.56  29.33  26.94 |
| T_1_ | 44.34  43.89  44.06 | 45.05  38.00  41.92 | 43.80  36.57  40.36 | 33.26  15.89  25.62 | 21.01  23.54  19.68 |
| T_2_ | 49.07  47.97  48.52 | 49.42  43.80  46.80 | 47.10  36.65  41.78 | 31.36  24.77  28.12 | 14.03  25.89  19.36 |
| T_3_ | 41.51  40.20  40.65 | 43.76  40.72  42.26 | 41.49  39.58  40.38 | 35.67  16.95  26.32 | 21.64  26.32  23.98 |
| T_4_ | 52.20  52.44  51.96 | 51.43  50.42  53.21 | 49.42  38.59  44.36 | 43.48  37.96  70.72 | 28.62  34.52  31.59 |

**Figure 5 Data Table: Effect of aloe-vera coating vitamin C at refrigerated condition**

| **Treatment** | **Storage period (Day)** | | | | |
| --- | --- | --- | --- | --- | --- |
|  | D_0_ | D_7_ | D_14_ | D_21_ | D_28_ |
| T_0_ | 168.2  166.3  167.9 | 152.4  155.9  154.3 | 146.5  147.6  147.05 | 97.9  95  96.3 | 68.4  72.6  70.5 |
| T_1_ | 162.5  165.9  164.2 | 158.6  162.4  160.5 | 147.8  141.2  144.5 | 137.2  145.6  141.4 | 112.2  117.9  115.05 |
| T_2_ | 166.6  172.6  168.9 | 161.3  158.6  158.8 | 164.2  159.6  161.7 | 141.6  151.6  146.3 | 124.4  129.7  127.05 |
| T_3_ | 162.2  157.8  159.7 | 160.4  176.3  168.3 | 152.0  143.6  147.2 | 148.6  146.5  147.5 | 126.8  132.1  128.6 |
| T_4_ | 172.5  168.4  169.7 | 167.6  162.5  165.06 | 167.8  157.9  161.3 | 155.0  157.8  156.2 | 136.9  145.9  141.9 |
