# Supplementary material for: Physical and chemical properties of aloe-vera coated guava (Psidium guajava) fruit during refrigerated storage
Source: PLoS One. 2023 Nov 1;18(11):e0293553. doi: 10.1371/journal.pone.0293553 (PMC10619840; doi:10.1371/journal.pone.0293553)
Supplement: S2 File — (DOCX) [file pone.0293553.s002.docx]

| Treatment | Day | | | | |
| --- | --- | --- | --- | --- | --- |
| a value | D_0_ | D_7_ | D_14_ | D_21_ | D_28_ |
| T_0_ | -7.41  -10.32 | -15.92  -35.88 | -10.98  -52.61 | -32.57  -21.67 | -19.4  -11.7 |
| T_1_ | -5.19  -8.09 | -15.09  -15.83 | -16.07  -26.84 | -8.15  -15.92 | -13.07  -4.46 |
| T_2_ | -7.46  -10.50 | -18.42  -21.42 | -17.87  -41.76 | -15.45  -10.87 | -13.53  -14.46 |
| T_3_ | -4.58  -7.37 | -15.45  -27.67 | -5.39  -21.78 | -19.58  -20.70 | -10.87  -10.34 |
| T_4_ | -6.58  -9.59 | -8.46  -8.15 | -18.42  -31.86 | -35.83  -28.40 | -16.67  -15.53 |
| B value |  |  |  |  |  |
| T_0_ | 32.37  32.94 | 9.62  10.70 | 15.67  14.03 | 12.19  16.42 | 10.38  16.89 |
| T_1_ | 28.14  28.55 | 9.81  22.15 | 29.56  23.10 | 31.57  18.36 | 16.66  20.23 |
| T_2_ | 31.27  31.84 | 15.67  18.86 | 12.19  18.41 | 16.42  15.19 | 12.79  23.10 |
| T_3_ | 27.07  28.76 | 31.57  33.36 | 32.79  15.45 | 15.52  24.56 | 17.78  16.89 |
| T_4_ | 31.84  31.45 | 19.33  19.58 | 16.80  18.41 | 21.58  15.15 | 18.41  17.08 |
| Hue angle |  |  |  |  |  |
| T_0_ | -77.10  -72.60 | -31.14  -16.60 | -55.03  -14.92 | -20.51  -37.15 | -48.17  -55.28 |
| T_1_ | -75.15  -74.14 | -33.02  -54.44 | -61.41  -40.69 | -75.52  -48.99 | -51.88  -77.56 |
| T_2_ | -56.58  -71.74 | -40.38  -41.44 | -34.31  23.89 | -43.76  -54.41 | -43.38  -57.95 |
| T_3_ | -80.43  -75.62 | -63.92  -50.32 | -80.66  -35.35 | -38.40  -49.87 | -58.56  -58.52 |
| T_4_ | -78.32  -73.04 | 66.36  67.40 | -42.36  -30.02 | -31.06  -28.07 | -47.83  -47.72 |

**Table 1 Data: Comparison of different color parameter (a, b and Hue values)**

**Table 2 Data: Proximate composition of guava and aloe-vera**

| Sample | Composition | | | | | | |
| --- | --- | --- | --- | --- | --- | --- | --- |
|  | Moisture | Protein | Fat | Carbohydrate | Ash | TSS | pH |
| Guava | 83.73  84.46  83 | 1.3  1.26  1.34 | - | 12.6  12.84  12.36 | 1.70  1.54  1.86 | 19.05  18.15  18.6 | 4.63  4.42  4.84 |
| Aloe vera | 97.4  98.6  96.92 | - | - | 1.77  1.63  1.7 | 1.38  1.3  1.22 | 1.2  1.56  0.84 | 4.76  4.60  4.92 |

**Table 3 Data: Effect of coating on total phenol content of Guava (mg GAE per 100 g)**

| Treatment | Storage periods (Day) | | | | |
| --- | --- | --- | --- | --- | --- |
|  | D_0_ | D_7_ | D_14_ | D_21_ | D_28_ |
| T_0_ | \| 265.7 \| \| --- \| \| 264.9 \| | \| 258.9 \| \| --- \| \| 252.3 \| | \| 192.3 \| \| --- \| \| 194.9 \| | \| 176.8 \| \| --- \| \| 168.6 \| | \| 138.3 \| \| --- \| \| 129.4 \| |
| T_1_ | \| 258.3 \| \| --- \| \| 254.6 \| | \| 245.6 \| \| --- \| \| 239.8 \| | \| 210.7 \| \| --- \| \| 212.6 \| | \| 194.2 \| \| --- \| \| 199.3 \| | \| 176.9 \| \| --- \| \| 169.4 \| |
| T_2_ | \| 261.4 \| \| --- \| \| 265.1 \| | \| 255.1 \| \| --- \| \| 248.6 \| | \| 232.4 \| \| --- \| \| 228.7 \| | \| 245.7 \| \| --- \| \| 232.8 \| | \| 192.8 \| \| --- \| \| 187.9 \| |
| T_3_ | \| 251.6 \| \| --- \| \| 246.8 \| | \| 245.2 \| \| --- \| \| 238.9 \| | \| 228.6 \| \| --- \| \| 221.4 \| | \| 218.9 \| \| --- \| \| 206.4 \| | \| 196.4 \| \| --- \| \| 192.4 \| |
| T_4_ | \| 269.8 \| \| --- \| \| 272.4 \| | \| 258.6 \| \| --- \| \| 255.3 \| | \| 243.9 \| \| --- \| \| 246.7 \| | \| 232.7 \| \| --- \| \| 228.1 \| | \| 217.9 \| \| --- \| \| 221.3 \| |
