# Supplementary figures and images for: Physical and chemical properties of aloe-vera coated guava (Psidium guajava) fruit during refrigerated storage
Source: PLoS One. 2023 Nov 1;18(11):e0293553. doi: 10.1371/journal.pone.0293553 (PMC10619840; doi:10.1371/journal.pone.0293553)

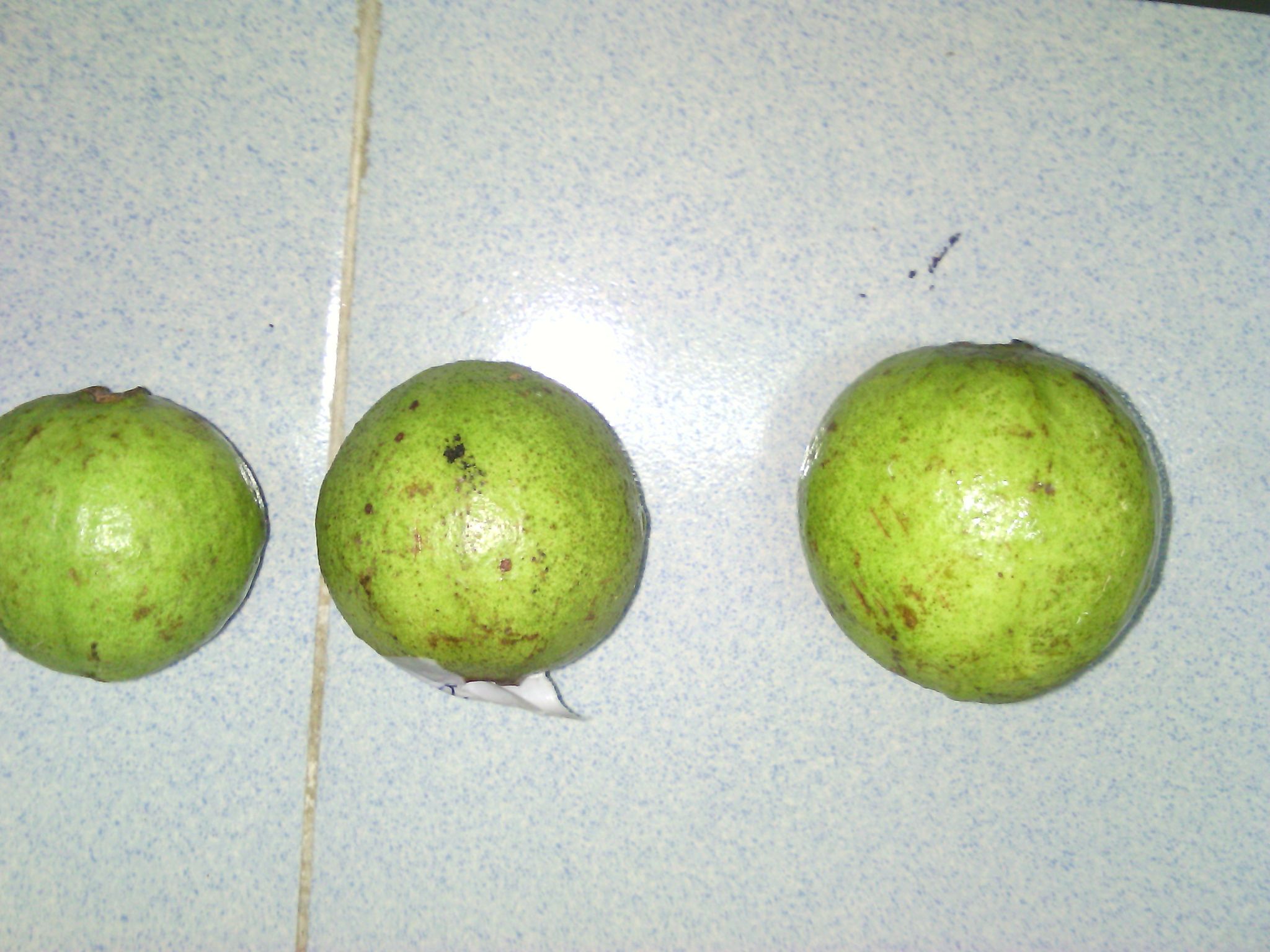

Supplement: S1 Fig — (JPG) [file pone.0293553.s003.jpg]
