# Supplementary material for: Physical and chemical properties of aloe-vera coated guava (Psidium guajava) fruit during refrigerated storage
Source: PLoS One. 2023 Nov 1;18(11):e0293553. doi: 10.1371/journal.pone.0293553 (PMC10619840; doi:10.1371/journal.pone.0293553)
Supplement: Attachment — Submitted filename: PONE-D-23-12719_reviewer.pdf-11.pdf [file pone.0293553.s007.pdf]

PLOS ONE

Physical and chemical properties of Aloe vera coated guava (Psidium guajava) fruit during refrigerated storage.

--Manuscript Draft--

Manuscript Number:	PONE-D-23-12719
Article Type:	Research Article
Full Title:	Physical and chemical properties of Aloe vera coated guava (Psidium guajava) fruit during refrigerated storage.
Short Title:	Physical and chemical properties of Aloe vera coated guava
Corresponding Author:	Md. Asaduzzaman, M. Engg.<br>State University of Bangladesh<br>Dhaka, BANGLADESH
Keywords:	Physical; Chemical; Coating; Guava; Refrigeration
Abstract:	Various concentration of aloe vera based coating formulation (25%, 50%, 75%, and 100%) was applied on fresh whole guava by dipping method. The guava was stored at a refrigerated condition (40C) and weight loss, color, firmness, vitamin C, total phenol and pH change were observed in this research. A significant effect of aloe vera coating was found over the storage period. Aloe vera treatment lowered the weight loss, also retard the texture and color than the control sample throughout 28 days of storage periods. Vitamin C and total phenol content seemed highest value 141.4 mg/100g and 219.6 mg GAE/100g respectively in 100% aloe vera coated sample after 28 days of storage than the control. Among 25%, 50%, 75%, and 100% aloe vera based coating sample, 100% aloe vera was best coating material to prevent the physical changes in fresh guava.
Order of Authors:	Debashis Kumar Dutta Roy, Masters of Science
	Tanny Saha, Masters of Science
	Nazma Khatun, Masters of Science
	Md. Asaduzzaman, M. Engg.
Additional Information:	
Question	Response
**Financial Disclosure**<br><br>Enter a financial disclosure statement that describes the sources of funding for the work included in this submission. Review the submission guidelines for detailed requirements. View published research articles from *PLOS ONE* for specific examples.<br><br>This statement is required for submission and **will appear in the published article** if the submission is accepted. Please make sure it is accurate.	The author(s) received no specific funding for this work.

Powered by Editorial Manager® and ProduXion Manager® from Aries Systems Corporation

**Unfunded studies**<br>Enter: *The author(s) received no specific funding for this work.*<br><br>**Funded studies**<br>Enter a statement with the following details:<br>• Initials of the authors who received each award<br>• Grant numbers awarded to each author<br>• The full name of each funder<br>• URL of each funder website<br>• Did the sponsors or funders play any role in the study design, data collection and analysis, decision to publish, or preparation of the manuscript?<br>• **NO** - Include this sentence at the end of your statement: *The funders had no role in study design, data collection and analysis, decision to publish, or preparation of the manuscript.*<br>• **YES** - Specify the role(s) played.<br><br>* typeset	
**Competing Interests**<br><br>Use the instructions below to enter a competing interest statement for this submission. On behalf of all authors, disclose any competing interests that could be perceived to bias this work—acknowledging all financial support and any other relevant financial or non-financial competing interests.<br><br>This statement is required for submission and **will appear in the published article** if the submission is accepted. Please make sure it is accurate and that any funding sources listed in your Funding Information later in the submission form are also declared in your Financial Disclosure statement.<br><br>View published research articles from *PLOS ONE* for specific examples.	The authors have declared that no competing interests exist.

**NO authors have competing interests**<br><br>Enter: *The authors have declared that no competing interests exist.*<br><br>**Authors with competing interests**<br><br>Enter competing interest details beginning with this statement:<br><br>*I have read the journal's policy and the authors of this manuscript have the following competing interests: [insert competing interests here]*<br><br>* typeset	
**Ethics Statement**<br><br>Enter an ethics statement for this submission. This statement is required if the study involved:<br><br>• Human participants<br>• Human specimens or tissue<br>• Vertebrate animals or cephalopods<br>• Vertebrate embryos or tissues<br>• Field research<br><br>Write "N/A" if the submission does not require an ethics statement.<br><br>General guidance is provided below. Consult the submission guidelines for detailed instructions. **Make sure that all information entered here is included in the Methods section of the manuscript.**	n/a

**Format for specific study types**<br><br>**Human Subject Research (involving human participants and/or tissue)**<br>• Give the name of the institutional review board or ethics committee that approved the study<br>• Include the approval number and/or a statement indicating approval of this research<br>• Indicate the form of consent obtained (written/oral) or the reason that consent was not obtained (e.g. the data were analyzed anonymously)<br><br>**Animal Research (involving vertebrate animals, embryos or tissues)**<br>• Provide the name of the Institutional Animal Care and Use Committee (IACUC) or other relevant ethics board that reviewed the study protocol, and indicate whether they approved this research or granted a formal waiver of ethical approval<br>• Include an approval number if one was obtained<br>• If the study involved *non-human primates*, add *additional details* about animal welfare and steps taken to ameliorate suffering<br>• If anesthesia, euthanasia, or any kind of animal sacrifice is part of the study, include briefly which substances and/or methods were applied<br><br>**Field Research**<br><br>Include the following details if this study involves the collection of plant, animal, or other materials from a natural setting:<br>• Field permit number<br>• Name of the institution or relevant body that granted permission	
**Data Availability**<br><br>Authors are required to make all data underlying the findings described fully available, without restriction, and from the time of publication. PLOS allows rare exceptions to address legal and ethical concerns. See the PLOS Data Policy and FAQ for detailed information.	Yes - all data are fully available without restriction

A Data Availability Statement describing where the data can be found is required at submission. Your answers to this question constitute the Data Availability Statement and **will be published in the article**, if accepted.<br><br>**Important:** Stating 'data available on request from the author' is not sufficient. If your data are only available upon request, select 'No' for the first question and explain your exceptional situation in the text box.<br><br>Do the authors confirm that all data underlying the findings described in their manuscript are fully available without restriction?	
**Describe where the data may be found in full sentences. If you are copying our sample text, replace any instances of XXX with the appropriate details.**<br><br>• If the data are **held or will be held in a public repository**, include URLs, accession numbers or DOIs. If this information will only be available after acceptance, indicate this by ticking the box below. For example: *All XXX files are available from the XXX database (accession number(s) XXX, XXX.).*<br>• If the data are all contained **within the manuscript and/or Supporting Information files**, enter the following: *All relevant data are within the manuscript and its Supporting Information files.*<br>• If neither of these applies but you are able to provide **details of access elsewhere**, with or without limitations, please do so. For example:<br><br>*Data cannot be shared publicly because of [XXX]. Data are available from the XXX Institutional Data Access / Ethics Committee (contact via XXX) for researchers who meet the criteria for access to confidential data.*<br><br>*The data underlying the results presented in the study are available from (include the name of the third party*	n/a

and contact information or URL).<br>• This text is appropriate if the data are owned by a third party and authors do not have permission to share the data.<br><br>* typeset	
Additional data availability information:	

Click here to access/download;Manuscript;Manuscript.docx

TITLE PAGE

Title: Physical and chemical properties of Aloe vera coated guava (Psidium guajava) fruit during refrigerated storage.

Author's information

1. Mr. Debashis Kumar Dutta Roy
Email: debashis.kdroy@gmail.com
Masters of Science
Department of Food Processing and Preservation,
Hajee Mohammad Danesh Science and Technology University, Dinajpur, Bangladesh

2. Mrs. Tanny Saha
Email: tanny14d@gmail.com
Masters of Science
Department of Food Processing and Preservation,
Hajee Mohammad Danesh Science and Technology University, Dinajpur, Bangladesh

3. Mst. Nazma Khatun
Email: najma.cnpi@gmail.com
Department of Food Technology,
Chapainawabganj Polytechnic Institute, Bangladesh.

Corresponding Author

4. Md. Asaduzzaman
Lecturer and academic coordinator,
Department of Food Engineering and Technology,
Sobhan Tower, 138, Mirpur Rd, Dhaka 1205, Bangladesh,
State university of Bangladesh.
Orcid ID: 0000-0001-7202-961X
Tel.: +8801710481107
E-mail: bitatipu@gmail.com

Core findings

- Aloe vera treatment lowered not only the weight loss but also the texture and color than the control sample throughout 28 days of storage periods.
- Vitamin C and total phenol content seemed highest value 141.4 mg/100g and 219.6 mg GAE/100g respectively in 100% aloe vera coated sample.
- Among 25%, 50%, 75%, and 100% aloe vera based coating sample, 100% aloe vera was best coating material to prevent the physical changes in fresh guava.

ABSTRACT

Various concentration of aloe vera based coating formulation (25%, 50%, 75%, and 100%) was applied on fresh whole guava by dipping method. The guava was stored at a refrigerated condition (4^0C) and weight loss, color, firmness, vitamin C, total phenol and pH change were observed in this research. A significant effect of aloe vera coating was found over the storage period. Aloe vera treatment lowered the weight loss, also retard the texture and color than the control sample throughout 28 days of storage periods. Vitamin C and total phenol content seemed highest value 141.4 mg/100g and 219.6 mg GAE/100g respectively in 100% aloe vera coated sample after 28 days of storage than the control. Among 25%, 50%, 75%, and 100% aloe vera based coating sample, 100% aloe vera was best coating material to prevent the physical changes in fresh guava.

Keywords: Physical, Chemical, Coating, Guava, Refrigeration

INTRODUCTION

Guava (*Psidiumguajava*) is a most common tropical fruit in Bangladesh. It is locally known as Peyaraor "Apple of Bengal". Basically guava is popular for its high content of vitamin C as 260 mg per 100 gm (Bose and Mitra, 2011). It is also rich in antioxidants which can retard aging by reducing oxidative damage of lipids, protein and nucleic acid (Feskanich*et al.*, 2000). According to Hossen,2012, the annual production of guava is about 145,000 m tons in an area of about 10,000 ha per year averagely and 30-40% of it is being wasted due to several causes in Bangladesh (Source: Directorate of Agriculture Marketing, Bangladesh Agriculture Develop Council (BADC).

Guava is highly perishable and its shelf-life ranges from 3 to 10 days at room temperature (Campbel, 1994). Without any treatment guava spoiled very first because of its high respiration rates, mechanical damage and microbial decomposition. Different types of method are used as ionizing radiation, preservatives and controlled atmosphere (Silva*et al.*,2011; Lima*et al.*,2003; Singh and Paul, 2008).

Additionally application of edible coating can increase the shelf life by reducing the moisture and gas loss. Edible coatings can improve the quality, safety, transportation, storage, and display of a wide range of fresh and processed fruits (Baldwin *et al.*, 1995). However, edible films and coatings also may prevent moisture loss and contamination of fruits and vegetables (Park, 1999). They can act as moisture and oxygen barrier during processing, handling, transportation (Díaz-Pérez *et al.*, 2001). During storage, it helps to retard decomposition and enhance the safety of guava by improving antimicrobial functions (Vargas *et al.*, 2008).In recent studies, edible coating formulation is used as a new improved technique. The thin film can act as a packaging material. However, it has more functional properties than thermoplastic materials (Brody, 2006).

Different types of edible coating are used and aloe vera is one of them. Aloe veragel is a polysaccharide based edible coating material,which has a commercial application in processing of fruits(Ahmed *et al.*, 2009). It has a therapeutic be an innovative and interesting means for commercial application and an alternative to the use of postharvest treatments. *Aloe vera*has been used for centuries for its medicinal and therapeutic, antioxidant and anti-microbial properties (Capasso*et al.*, 1998; Hamman, 2008; Chen *et al.*, 2007; and Miranda *et al.*, 2009). Various types of coating condition and different coating materials canenhance the shelf life different fruits(Chen, 2001).

Though a large quantity of guava is waste during post-harvest handling, coating of guava can reduce this type of loss. Also research work regarding aloe vera coating on guava was not carried in Bangladesh. Considering the above issues this research work was undertaken with the following objectives:

1. To find out suitable coating concentration of aloe vera for guava.
2. To analyse the effect of various concentrations of aloe vera on physical and chemical properties of fresh and coated guava over the storage period.

MATERIALS AND METHODS

Experimental site

The study was conducted in the laboratory of Food Engineering & Technology and Food Processing & Preservation department under the Faculty of Engineering of Hajee Mohammad Danesh Science and Technology University, Dinajpur. The study was also conducted in the chemistry laboratory of this university.

Samples and chemical collection

Fresh guava and Aloe vera leaves were bought from local market, Dinajpur. Guava fruits of uniform size and shape and without any defect were selected. All analytical grade chemicals such as Glycerol, methanol, Glacial acetic acid, Meta phosphoric acidwere supplied by the food processing laboratory, Hajee Mohammad Danesh Science and Technology University, Dinajpur.

Preparation of aloe vera gel

Aloe vera leaves were washed with water and skins were peeled. The gels were separated, collected and ground in a blender. Then aloe vera pulp was filtered to remove the fibres (Navarro *et al.*, 2011).

Process of making edible coating

Edible coating material was made by adding 1% glycerol (used as plasticising agent), 5% Corn starch (used as thickening agent), 5% ethanol (used for fast dring of coating), and 1% acitic acid with Aloe vera gel and stirred evenly for 20 minutes. Then the aloe vera gel solution was heated at a temperature of 70°C for 45 minutes for pasteurized it. The gel solution was then cooled to room temperature.

Guava coating process

The gel solutionof aloe vera was taken in a beaker. Fresh guavas then dipped into with different concentrations of aloe vera (T_0, T_1, T_2, T_3 and T_4) for 2 minutes and then dried naturally for 30 minutes where

T_0: control, untreated guava

T_1: guava was coated with 25% Aloe vera

T_2: guava was coated with 50% Aloe vera

T_3: guava was coated with 75% Aloe vera

T_4: guava was coated with 100% Aloe vera

After drying the coated guavas were packed using LDPE and stored refrigerated at 4°C for 30 days.

Physical properties

Determination of pH

The pH of the selected samples was determined by the conventional procedure by a pH meter. The electrode assembled of the pH meter was dipped into the standard buffer solution of pH 7.0 taken in a clean and dry beaker. The fine asymmetry potential knob was adjusted to pH 7.0, then in a same way at pH 4. After calibration the electrode assembled pH meter was dipped into the selected sample solution; the pH was then readout washed twice with distilled water. Again it was dipped into another sample to determine the pH.

Weight loss

Sample weight loss was determined by comparing the weights of coated guava after storage with initial weights and expressing the results as percentage (Chien*et al.*, 2007). The results calculated with following equation where W_1 is Initial weight and W2 is Final weight.

$$\text{Weight loss} = (W_1 - W_2)/ W_1 \times 100$$

Firmness

Firmness of Guava was determined by using a penetrometer. The firmness test was done by penetrating the stainless steel probe with 3.5 mm of diameter. The probe was penetrating up to at three different locations in each sample. The data was recorded as a force express as unit of Newtons (N).

Color

The color property of guava was determined with a Colorimeter Minolta CM-2500d (Konica Minolta Optics, Inc. Japan). Color attributes were recorded as L* (lightness), a* (redness) and b* (yellowness). Here the change in lightness and Hue angle is calculated. The equation used for hue is as follows where h = Hue angle, b= yellowness and a= redness

$$h = \tan^{-1} (b/a).$$

Chemical properties

Proximate composition

Moisture, crude protein, fat and ash content of flours were determined by official methods (AOAC, 1998)

Vitamin C

Vitamin C content of guava was measured using AOAC method (2,6-Dichloroindophenol titrimetric method, 2006).

Total phenol

Total phenol was determined according to the method described by Lin JY, 2007*et al*. with slight modification. Carrot jam (1g) was extracted with 20 ml of methanol and concentrated up to 10 ml by hot plate at 60ºC. Again, volume was adjusted to 20 ml with methanol and filtered through whatman no.1. After that 1ml sample, 0.2ml of 10% Folin reagent was transferred to a test tube. After 3 minutes, 0.8 ml of 7.5% Na_2CO_3 was added and kept in dark for 1hr at room temperature. Then the absorbance was taken at 760 nm. The phenol content was determined using Gallic acid standard curve and was expressed as ppm.

Total phenol content was calculated by following formula:

$$\% \text{ Phenol} = \text{X (ppm)} \times \frac{\text{Total volume made up}}{\text{Weight of sample(mg)}} \times 100$$

Statistical Analysis

Each experiment included three replications. Data were analysed using statistical software (R-version 3.2.2.). A multifactorial analysis of variance was carried out. Individual effects of the factors have been calculated on a particular point of time during the study. Differences were considered to be significant at $P<0.05$.

RESULTS AND DISCUSTION

PHYSICAL PROPERTIES

pH

The pH content of guava was decreased gradually with the increasing of storage time. The result has shown in Figure 01. 100% aloe vera coated sample shows the lowest pH 3.62 at day 28 compared with the others with significantly different ($P>0.05$). That means 100% aloe vera more control the microbial growth of microbes, Miranda *et al.*, *(*2009). This pH change can be the organic acid substances or conversion of sugar into acid and metabolic processes, (Keditsu*et al.*,2003).

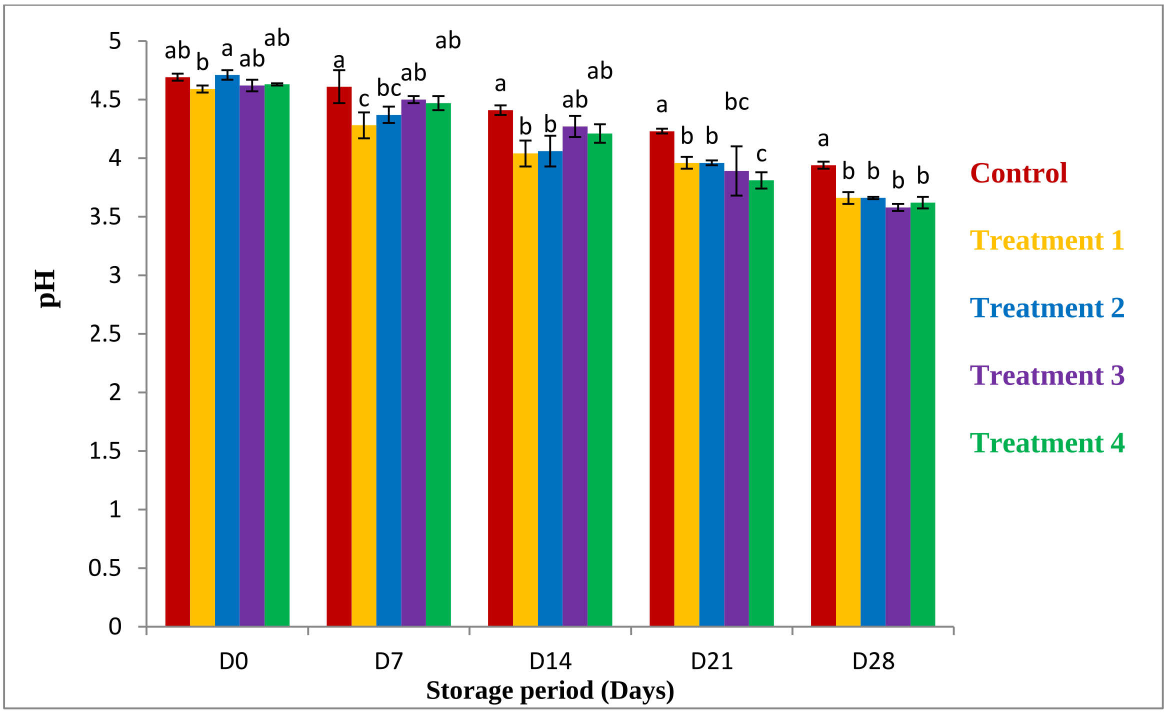

*Values are mean of triplicate analysis with standard mean error.

Figure 01: pH and storage period relationship

Weight loss

The weight loss was measured up to 35 days, here the data showed for 28 days in Figure 02. The change of weight loss was increased through the storage periods. Highest weight loss was found in the control sample compare with the coated one. The change is the control sample drastically high as 14.17% at 28 days and low as 6.36 in 100% aloe vera coated sample. However different amount of weight loss was observed in different treatment. The weight loss was minimized by aloe vera coating. The concentration of different aloe vera juice is caused that Dorria (2007). The result we found is nearly same to Zuraidah (2015). The weight loss takes place because of dehydration, respiration. The physical cell disruption is alsoone of the causes of weight (Vazquez-Ochoa, 1990).

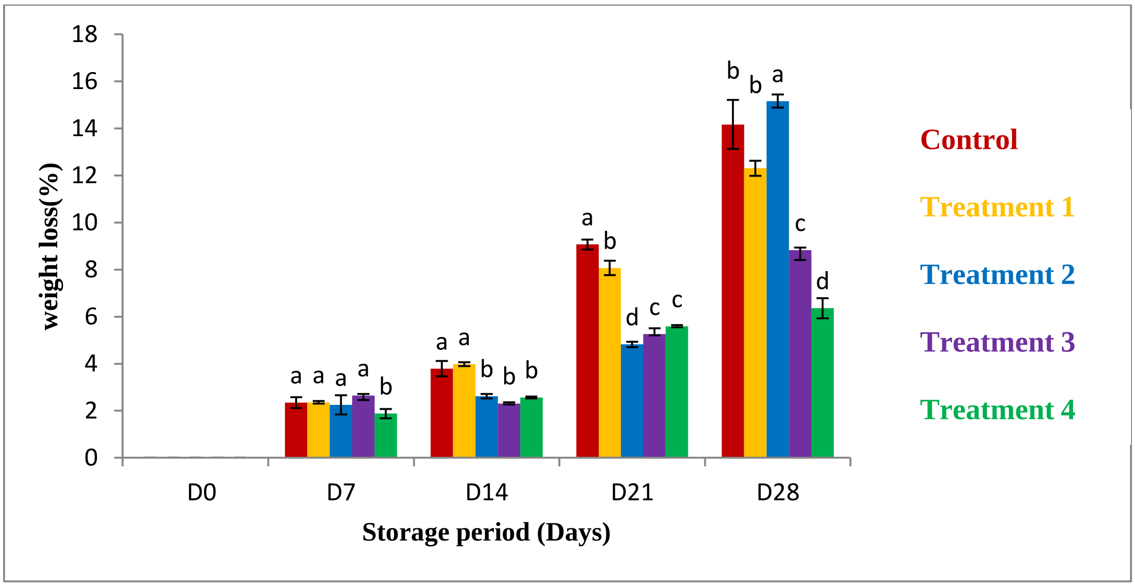

*Values are mean of triplicate analysis with standard mean error.

Figure 02: Effect of coating on Weight loss

Firmness

Weight loss can affect the firmness of guava. Softness of guava also affects by ethylene activity and in room temperature it changes within 4 days at room temperature (Azzoliniet *al*., 2005; Brown & Wills, 1983). Figure 03 represents that the firmness is gradually lower by increasing the storage period. In case of coated sample the firmness is higher than the uncoated ones and 100% aloe vera coating restrict the firmness loss at the 28 days of storage period. Martinez –Romero *et al.*, (2006) also found that aloe vera gel

has a power to lower the weight loss which also influences the retarding of firmness. The result has little bit variation to Zuraidahet *al*.(2015).The possible reason may be the use of higher concentration of aloe vera in this study. The higher concentration of aloe vera retards the firm loss and improved quality.

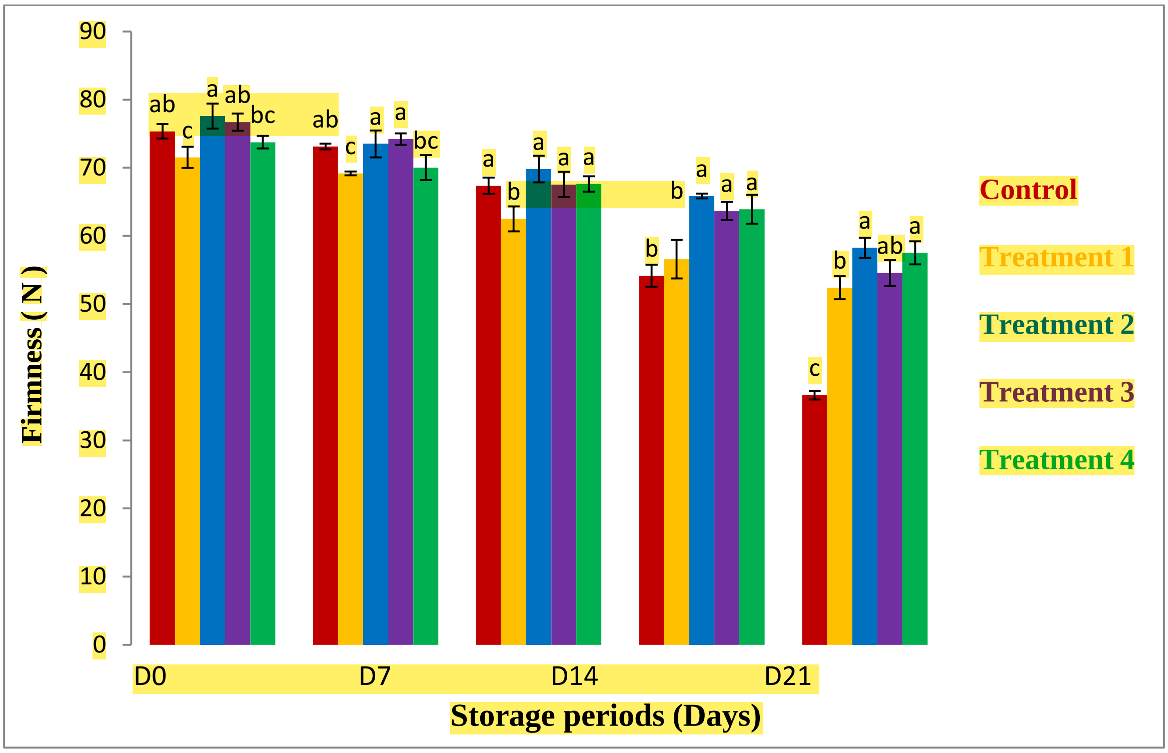

*Values are mean of triplicate analysis with standard mean error.

Figure 03: Changes in Firmness

Color attribute of guava

The change in lightness, redness, and yellowness are shown in figure 04 and Table 01. The value showed negative in case of a, because guava is generally green in color. When storage time was increased lightness of guava decreased (highest 47.15 to 26.94 in controlled and 52.32 to 31.57 in 100% aloe vera coated sample). But the coated sample has higher lightness than uncoated sample. At day 28, 100% aloe vera coated sample had highest value than others. One of the possible reasons of change in lightness is coating material. It acts as a modified atmosphere packaging materials which delay the degradation of color (Ergun and Satici, 2012).According to Baldwin *et al.* (1995) edible coating also delay color change, weight loss in tropical fruit and improve appearance.

In case of green ness, it was lowered throughout the storage period. The green color changed to light yellow to dark yellow. The controlled sample changed its color very first

because of its high respiration rate than the other coated sample. Previous study also showed the same result, (Siqueira*et al.*, 2011; González*et al.*, 2011).

With the change of time the hue angles were increased that shown in the Table 2. This indicates also the changing of green to yellow color. The reduction of greenness (-8.08 to -16.01) is nearly similar to Xing et al. (2011), who used a poly saccharide based chitosan coating.

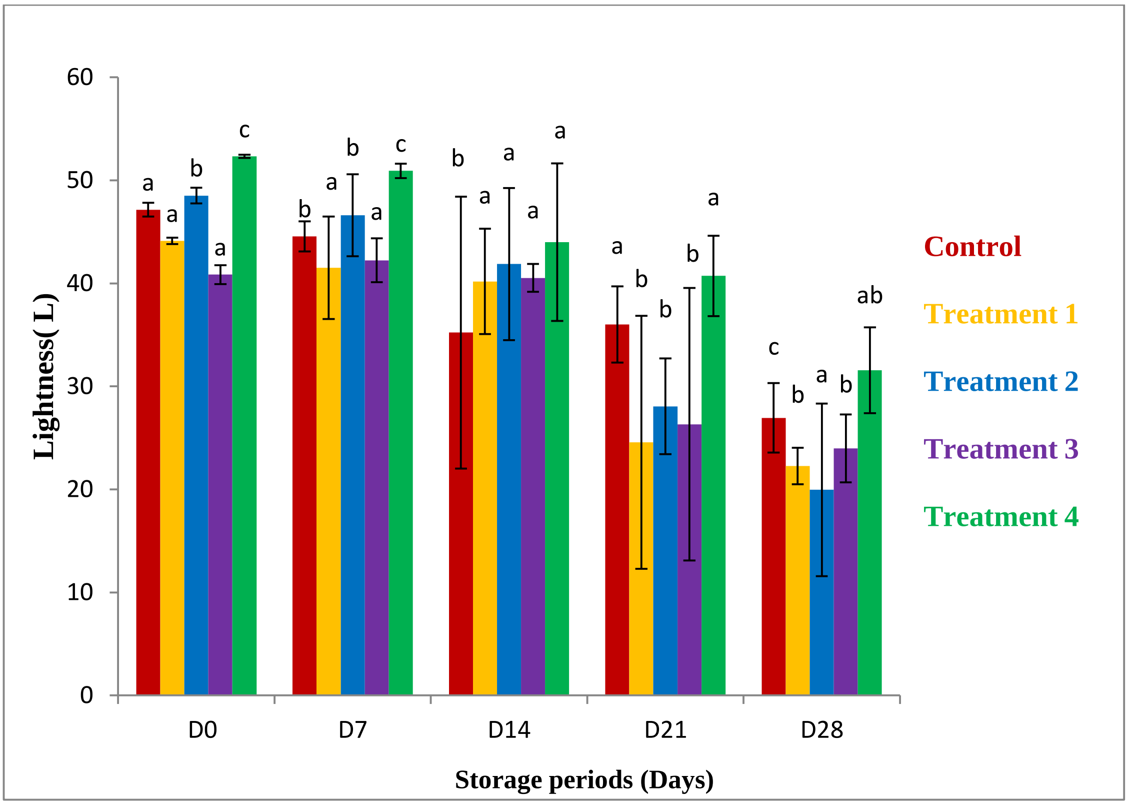

*Values are mean of triplicate analysis with standard mean error.

Figure 04: Effect of coating on lightness

Table 01: a, b and Hue values of the product

Treatment	Storage periods (Days)				
	D_0	D_7	D_{14}	D_{21}	D_{28}
**a value**					
T_0	-8.86 ± 2.05^a	-25.9 ± 1.41^a	-31.79 ± 2.94^c	-27.12 ± 7.70^b	-3.85 ± 2.11^b
T_1	-6.64 ± 2.05^a	-15.46 ± 0.5^{ab}	-21.45 ± 7.61^a	-12.03 ± 5.41^a	-4.30 ± 1.23^b
T_2	-8.98 ± 2.14^a	-19.92 ± 2.1^{ab}	-29.81 ± 1.7^{ab}	-13.16 ± 3.23^a	-13.99 ± 0.65^a

T_3	-5.97±1.91^{a}	-21.56±8.64^{a}	-13.5±1.54^{ab}	-20.1±0.79^{ab}	-10.60±0.37^{b}
T_4	-8.08±2.12^{a}	-8.30±0.21^{a}	-25.14±9.5^{b}	-32.11±5.25^{b}	-16.1±0.81^{b}
**b value**					
T_0	32.65±0.40^{a}	9.54±1.41^{b}	14.85±1.15^{ab}	14.30±2.99^{b}	13.63±4.6^{b}
T_1	28.34±0.28^{b}	6.17±2.25^{b}	26.33±4.56^{a}	24.96±9.34^{a}	18.44±2.52^{a}
T_2	31.55±0.40^{a}	17.26±2.25^{c}	3.11±2.16^{ab}	15.805±0.86^{b}	17.94±7.21^{a}
T_3	27.91±1.19^{b}	32.46±1.26^{a}	24.12±1.22^{a}	20.04±6.39^{a}	17.33±0.62^{a}
T_4	31.64±0.27^{a}	19.45±0.17^{c}	0.80±2.48^{b}	18.36±4.54^{a}	17.74±0.94^{a}
**Hue angle (h^0)**					
T_0	-74.85±3.14^{a}	-23.87±1.02^{b}	-34.97±2.83^{a}	-28.83±1.17^{a}	-21.72±5.02^{c}
T_1	-74.64±0.71^{a}	-43.73±1.5^{bc}	-51.05±1.4^{ab}	-62.255±1.8^{b}	-64.72±1.81^{b}
T_2	-64.1±1.07^{ab}	-40.9±0.74^{bc}	-52.1±4.1^{b}	-49.08±7.5^{ab}	-50.66±1.03^{a}
T_3	-78.02±3.40^{b}	-57.12±9.61^{c}	-58±3.21^{ab}	-44.13±8.1^{ab}	-58.54±0.03^{ab}
T_4	-75.68±3.73^{a}	-66.88±0.73^{a}	-36.19±8.72^{a}	-29.56±2.11^{a}	-47.77±0.08^{a}

*Values are mean of triplicate analysis with standard mean error
*Means with the same column with different letters are significantly different ($P<0.05$)
*T_0= Control, T_1 = 25% Aloe vera, T_2 =50% Aloe vera,T_3 =75% Aloe vera, T_4 =100% Aloe vera.

CHEMICAL PROPERTIES

Proximate analyses

The chemical composition of guava and aloe vera has shown in Table 02. In case of guava the result was found 83.73% moisture, 12.6% carbohydrate, 1.7% ash and 1.3% protein which nearly samereported by USDA, 1982 and Khatun, 2011. They found 80.61% moisture, 0.7% ash, 1.28% acidity, 11%carbohydrate, 19% TSS. The variation in the result may because of the different species and environmental and horticultural condition of guava production; Yusof (1990). On the other hand, in case of aloe vera the result shows 97.4% moisture, 1.3% ash and 1.7% carbohydrate. Pierce (1983) and Rowe (1941) also found 98.5%moisture and 0.3% carbohydrate which are nearly similar.

Table 02: Proximate composition of guava and aloe vera

Sample	Composition (%)						
	Moisture	Protein	Fat	Carbohydrate	Ash	TSS	pH
Guava	83.73±0.73	1.3±0.04	-	12.6±0.24	1.7±0.16	18.6±0.45	4.63±0.21

Aloe vera	97.4±0.52	-	-	1.7±0.07	1.3±0.08	1.2±0.36	4.76±0.16

*Values are mean of triplicate analysis with standard mean error

Vitamin C

Vitamin C is an important factor for guava. The change in vitamin C of guava is shown in Figure 05. In this study, the result shows vitamin c content of raw guava was 167.25 mg/100g. The result is similar to Vila*et al.* (2007)168.5 mg/100g at day 1.

During storage period vitamin C content of guava is decreased in all samples. The loss of vitamin C in control sample is 96.75mg/100g and in coated sample were 49.46, 42.55, 30.55, 29.05mg/100g respectively. The result shows that vitamin C loss was higher in fresh sample than the coated sample. Vitamin C loss may occur because of phenol oxidase and ascorbic acid oxidase (Yaman and Bayoundurh, 2002). Also the presence of light, heat and oxygen can reduce the vitamin C content (Davey *et al.*, 2000).On the other hand modified packaging (aloe vera coating) creates an aerobic condition to the guava surface. Due to this oxidation of ascorbic acid is influenced and lowering the ascorbic acid content Lee and Kader, (2000).

This study concluded that the loss of vitamin C in aloe vera coated sample is lower than the controlled and higher the concentration of aloe vera has higher efficiency. Here 100% coating concentration shows higher result.Serrano *et al.* (2006) also found that aloe vera gel coating for grapes was able to control the ascorbic acid content loss during storage.

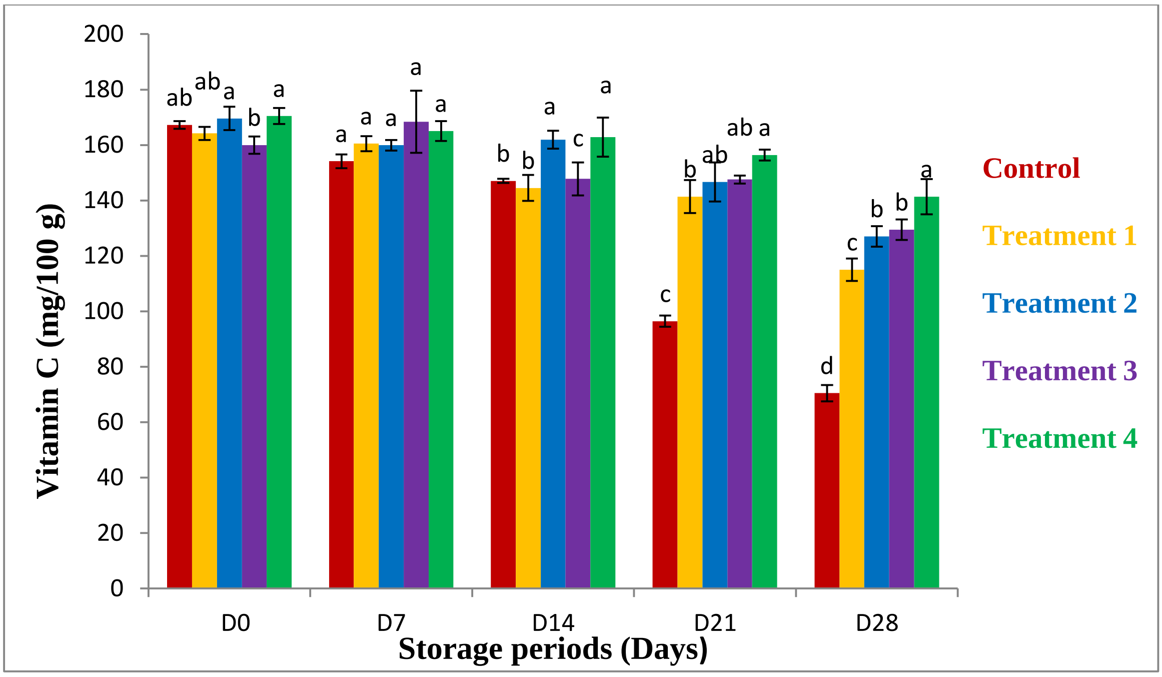

*Values are mean of triplicate analysis with standard mean error.

Figure 05: Vitamin C

Total phenol content

Total phenol is also an important factor for maturity index. Table 3 shows the change in total phenol throughout the storage periods. The result showed that total phenol content of guava in fresh sample was 265.3-271.1 mg GAE per 100 g. This result is similar to Thaipong, K. *et al.*(2006), and Hagen *et al.* (2007) who reported that it could be 170–344 mg GAE per 100g.

During the storage periods total phenol content is reduced 265.3 to 133.85 for control and 271.1 to 219.6 mg GAE per 100g for 100% aloe vera coated sample. The loss in control sample was 131.45 and in coated sample were 83.3, 72.9, 54.8 and 51.5 mg GAE per 100g respectively. The change may due to many factors as variety, species, ripening and environmental condition, Iqbal, et al. (2006). According to Taylor (1993), the lowering in total phenol content can reduce the astringency during storage period. More over some monomeric component can convert to polymeric components; these also cause the reduction of total phenol, (Iversen, 1999 Ochoa et al.,1999).

This study shows that the loss of total phenol is reduced by the application of aloe vera coating. This is also proven by Serrano *et al.* (2006).

Table 03: Effect of coating on total phenol (mg GAE per 100 g)

Treatment	Storage periods (Day)				
	D_0	D_7	D_{14}	D_{21}	D_{28}
T_0	265.3 ± 0.56^{ab}	255.6 ± 4.66^{d}	193.6 ± 1.83^{d}	172.7 ± 5.79^{c}	133.85 ± 6.29^{d}
T_1	256.45 ± 2.61^{c}	242.7 ± 4.10^{b}	211.65 ± 1.34^{c}	196.75 ± 3.60^{b}	173.15 ± 5.30^{c}
T_2	263.25 ± 2.62^{b}	251.85 ± 4.59^{ab}	230.55 ± 2.61^{b}	239.25 ± 9.12^{a}	190.35 ± 3.46^{b}
T_3	249.2 ± 3.39^{d}	242.05 ± 4.45^{b}	225 ± 5.09^{b}	212.65 ± 8.83^{b}	194.4 ± 2.82^{b}
T_4	271.1 ± 1.83^{a}	256.95 ± 2.33^{d}	245.3 ± 1.97^{a}	230.4 ± 3.26^{a}	219.6 ± 2.40^{a}

*Values are mean of triplicate analysis with standard mean error

Storage studies of guava

Organoleptic properties of all the samples of guava were observed during storage periods at refrigeration temperature (4^0C), on the basis of color, flavour, texture, and visual fungal growth. This was recorded at every 7 days interval up to 35 days. The data are shown in table 04. In case of control sample there was no change in the properties up to 7 days. At day 14 the color was staring to change in dark green to green and slightly fungal growth. At day 21 it was changed its all property and organolaptically unaccepted.

Table 04: Sensory studies of guava

Storage period	Sample	Color	Flavor	Texture	Visual fungal growth	Remarks
Day 0	T_0	Dark Green	Good	Hard	No growth	Good
	T_1	Dark Green	Good	Hard	No growth	Good
	T_2	Dark Green	Good	Hard	No growth	Good
	T_3	Dark Green	Good	Hard	No growth	Good
	T_4	Dark Green	Good	Hard	No growth	Good
Day 7	T_0	Dark Green	Good	Hard	No growth	Good
	T_1	Dark Green	Good	Hard	No growth	Good
	T_2	Dark Green	Good	Hard	No growth	Good
	T_3	Dark Green	Good	Hard	No growth	Good
	T_4	Dark Green	Good	Hard	No growth	Good
Day 14	T_0	Green	Good	Semi Soft	Slightly growth	Good
	T_1	Dark Green	Good	Semi Soft	No growth	Good
	T_2	Dark Green	Good	Semi Soft	No growth	Good
	T_3	Dark Green	Good	Semi Soft	No growth	Good
	T_4	Dark Green	Good	Semi Soft	No growth	Good

Day 21	T_0	Slight yellowish	Off flavor	Soft	Highly growth	Spoiled
	T_1	Green	Good	Semi Soft	No growth	Good
	T_2	Green	Good	Semi Soft	No growth	Good
	T_3	Green	Good	Semi Soft	No growth	Good
	T_4	Green	Good	Semi Soft	No growth	Good
Day 28	T_0	Fully yellowish	Off flavor	No texture	Excessive growth	Spoiled
	T_1	Slight yellowish	Off flavor	Soft	Excessive growth	Spoiled
	T_2	Slight yellowish	Off flavor	Soft	Excessive growth	Slightly Spoiled
	T_3	Slight yellowish	Off flavor	Semi Soft	Medium growth	Slightly spoiled
	T_4	Slight yellowish	Off flavor	Semi Soft	Medium growth	Slightly spoiled
Day 35	T_0	Black	Off flavor	No texture	Excessive growth	Spoiled
	T_1	Full yellow	Off flavor	No texture	Excessive growth	Spoiled
	T_2	Full yellow	Off flavor	Semi Soft	Excessive growth	Spoiled
	T_3	Full yellow	Off flavor	Semi Soft	Mediumgrowth	Spoiled
	T_4	Full yellow	Off flavor	Semi Soft	Mediumgrowth	Spoiled

*Values are mean of triplicate analysis with standard mean error
*Means with the same column with different letters are significantly different (P<0.05)
*T_0= Control, T_1 = 25% Aloe vera, T_2 =50% Aloe vera,T_3 =75% Aloe vera, T_4 =100% Aloe vera.

In case of coated sample there was no change up to 21 days only a little bit softness. At day 28, 25% and 50% aloe vera coated sample was unaccepted because it change it properties. On the other hand 75% and 100% coated sample had better quality than the other sample and organolaptically accepted up to 28 days. But the entire sample was spoiled at day 35. Kendall and Sofos (2007) found that if the sample processed properly and stored in cool dry place it will retain good quality up to several months.

CONCLUSION

This study was conducted to introduce a novel processing technique, edible coating by aloe vera gel on guava with the objectives to document the physic-chemical properties and find out the suitable concentration of coating during storage periods.

Fresh guava and aloe vera leaf was collected from local market. The coating method was done by dipping method. Four different concentration of aloevera gel was used as 25%, 50%, 75% and 100%. The quality and physico- chemical properties were done by different established method. Significant different (P<0.05) was found between the control and coated sample during 35days of storage period.

The salient points of the study are summarized below:

- The analyzedproximate composition of guava and aloe vera gel were83.73% moisture, 1.3% protein, 12.6% carbohydrate, 1.7% ash and 97.4% moisture, 1.2% ash, 0.7% carbohydrate respectively.
- The weight loss and soft texture was foundhigher than the coated sample.
- The vitamin C content of guava was higher (141.4mg) in coated sample than the fresh guava (70.5mg)at the 28th day of storage periods.
- The color change (yellowness) was also lower in coated sample and had a better appearance and acceptances.
- The total phenol content loss was also controlled by the application of coating material.

Therefore it is concluded that application of aloe vera coating can increase the shelf life of guava. Also the higher concentration of coating material has the higher quality. Among all the treatment 100% aloe vera coating was best. As it is increases the shelf life and lowering the loss of nutritional and quality parameter so it can be used as a natural preservation technique.

Author contributions: Conceptualisation and methodology M.A.; initial draft D.K.D.R. and T.S.; review and editing M.N.K. and M.A. All authors have read and agreed to the submitted version of the manuscript.

Conflict of interest: The authors declare that they have no known competing financial interests or personal relationships that could have appeared to influence the work reported in this paper.

Funding: This study did not receive any specific grant from funding agencies in the public, commercial, or non-for-profit sectors.

REFERENCES

Abeles, F.B., Morgan, P.W.andSaltveit, M.E. (1992). Ethylene in plant biology. Acad. Press.

Adetunji, C.O. (2008). The antibacterial activities and preliminary phytochemical screening of vernoniaamygdalina and Aloe vera against some selected bacteria. M.sc Thesis.University of Ilorin.40-43.

Ahmed, M.J., Singh, Z., and Khan, A.S. (2009). Postharvest Aloe vera gel- coating modulates fruit ripening and quality of ‘Arctic Snow’nectarine kept in ambient and cold storage. *International journal of food science & technology,*44(5): 1024-1033.

AOAC (1998). Official Methods of Analysis of AOAC International (14th ed.). Washington, DC, USA: Association of Official Analytical Chemistry. Methods, 950.46, 938.08, 960.39, 955.04

Azzolini, M., Jacomino, A.P., Bron, I.U., Kluge, R.A., andSchiavinato, M. (2005). Ripening of “Pedro Sato” guava: Study on its climacteric or non-climacteric nature. *Brazilian Journal of Plant Physiology,* 17, 299–306.

Baldwin, E.A., Nisperos- Carriedo, M.O. and Baker R.A. (1995). Use of edible coatings to preserve quality of lightly (and slightly) processed products. *Critical Reviews in FoodScience & Nutrition,*35(6): 509-524.

Bose, T.K. (2011). Fruits of India, Tropical and Subtropical.1st edn, NayaProksash, Calcata – 6 India. 278.

Brecht, J.K. (1995). Physiology of lightly processed fruits and vegetables.*Horticultural Science,* 30:18-22.

Brody A., (2006). Nano and food packaging technologies converge. *Food Technology,* 60(3):92– 4.

Brown, B.I., and Wills, R.B.H. (1983). Post-harvest changes in guava fruit of different maturity. *ScientiaHorticulturae*, 19, 237–243.

Campbell, C.A. (1994). “Handling of Florida-grown and imported tropical fruits and vegetables,” *Horticultural Science*, 29: 975-978.

Capasso, F., Borrelli, F., Capasso, R., Di Carlo, G., Izzo, AA., Pinto, L., Mascolo, N., Castaldo, S., and Longo, R (1998). Aloe and its therapeutic use.*Phytotherapy Research,*12: 124-127.

Chen, SH., Lin, KY., Chang, CC., Fang, CL. and Lin, CP. (2007). Aloe-emodin- induced apoptosis in human gastric carcinoma cells. *Food and Chemical Toxicology,* 45: 2296-2303.

Cheow, C.S., Norizzah, A.R., Halimahton, M.S., Adi, M.S. andZahid, A. Mohd (2011). Optimization of process conditions for the application of edible coating emulsion onguava (Psidiumguajava) using response surface methodology. *IACSIT Press, Singapore*,7.

Chien, P.J., Sheu, F., and Yang, F.H. (2007).Effects of edible chitosan coating on quality and shelf life of sliced mango fruit.*Journal of Food Engineering*, 78(1): 225-229.

Davey, M.W., Van Montagu, M., Inzé, D., Sanmartin, M., Kanellis, A. and Smirnoff, N. (2000). Plant L-ascorbic: Chemistry, function, metabolism, bioavailable and effects of processing. *Journal of the Science of Food and Agriculture*, 80(7): 825–860.

Díaz-Pérez, JC.,Mejía, A., Bautista, S., Zavaleta, R., Villanueva, R. and Gómez, RL (2001). Response of sapotemamey [Pouteriasapota (Jacq.) HE Moore and Stearn] fruit to hot water treatments. *Postharvest biology and technology*,22(2):159-167.

Directorate of Agriculture Marketing, Bangladesh Agriculture Develop Council (BADC), Khamar Bari, Dhaka.

Domingo Martínez-Romero, Salvador Castillo, FabiánGuilléna,Huerts M. Díaz-Mula, Pedro J. Zapata, Daniel Valero, María Serrano (2013). Aloe vera gel coating maintains quality and safety of ready-to-eatpomegranate arils. *Postharvest Biology and Technology*,86:107–112.

Dorria, M.M.A. (2007). Jojoba oil as a noval coating for exported Valancia orange fruit, Part ii. The use of oil emulsion.*Americ Eurasian Journal of Agriculture and Environment Science*, 2(3): 261-267.

Ecocrop (2015).Ecocorp, database, FAO, Rome, Italy. (http://ecocrop.fao.org/ecocrop/svr/en/home).

El Boushy, A.R.Y., van der Poel, A.F.B., (2000). Handbook of poultry feed from waste: processing and use. *Springer-Verlag New York*, 428.

El Ghaouth A, Arul J., Ponnampalam, R., and Boulet, M. (1992b). Chitosan coating to extend the storage life of tomatoes.*Horticulture Science.* 27(9): 1016-1018.

El-Buluk, R., Babiker E.E.E. and El-Tiany, A.H. (1995).Bio-chemical and physical changes in fruits of our guava cultivars during growth and development.*Food chemistry,*54 (93) 279-282.

Ergun, M. and Satici, F. (2012).Use of *Aloe vera*gel as biopreservatives for ‘Granny Smith’ and‘Red Chief’ apples.*The Journal of Animal and Plant Science,*22:363–368.

Eshun K and He Q. (2004). Aloe vera: A valuable ingredient for the food, pharmaceutical and cosmetic industries—A review. *Critical Review Food Science Nutrition,* 44:91–6.

Esteves – MIF-Chitavra; Chitavra AB and Mb- Paula De.(1984). Characteristics of fruits of six guava (*Psuidiumguajava*L) cultivars during ripening.*Anais do VII congress porasilero de Furitivultura*, 2: 477-489.

Esua, M F. andRauwald, J W. (2006). Novel bioactive maloylglucans from *Aloe vera* gel: isolation, structure elucidation and in vitro bioassays. *Carbohydrate Resource,*27:355–364.

Feskanich, D., Ziegler, R.G., Michaud, D.S., Giovannucci, E.L., Speizer, F.E., Willett, W.C., andColditz, G.A. (2000).Prospective study of fruit and vegetable consumption and risk of lung cancer among men and women.*Journal of the National Cancer Institute*, 92(22): 1812–1823.

González, I.A., Osorio, C., Meléndez-Martínez, A.J., González-Miret, M.L., and Heredia, F.J. (2011). Application of tristimuluscolorimetry to evaluate colour changes during the ripening of colombian guava (Psidiumguajava L.) varieties with different carotenoid pattern. *International Journal of Food Science and Technology,* 46: 840–848.

Hagen, S.F., Borge, G.I.A., Bengtsson, G.B., Bilger,W., Berge, A. andHaffner, K. *et al.* (2007). Phenolic contents and other health and sensory related properties of apple fruit (MalusdomesticaBorkh., cv. Aroma): Effect of postharvest UV-B irradiation. *Postharvest Biology and Technology*, 45: 1–10.

Hamman, J.H. andViljoen, A.M. (2008).Use of Aloe vera for increasing the bioavailability of poorly absorbable drugs.*SA patent application,* 2008/01542.

Hossen, S.M. (2012). Statistical Analysis of Current Guava Production and Its Prospect in Bangladesh.*Asian Business Review,* 1(1):9-14.

Iqbal, S. andBhanger, M.I.(2006). Effect of season and production location on antioxidant activity of *Moringaoleifera*leaves grown in Pakistan. *Journal of Food Composition and Analysis, 19*: 544–551.

Islam, M.N., Bhuyan, M.A.J., Biswas, M. and Islam, M.S.(1993).Studies on processing and storage of fruits.Dept. of Food Technology and Rural industries, Bangladesh Agriculture University, Mymensingh, May, 1993.

Iversen, CK. (1999). Black currant nectar: effects of processing and storage on anthocyanin and ascorbic acid content. *Journal of Food Science,*64:37–41.

Keditsu, S.E., Smith, S.T. and Gomez, J. (2003).Effect on ethanol vapor treatments on light brown apple.*Postharvest Biology and Technology*, 18: 268-278.

Kendall, P. and Sofos, J. (2007).Leathers and Jerkies, Food and Nutrition Series, Fact Sheet no. 9.311,Colorado State University Extension, U.S.

Khatun, R. (2011). Studies on storage stability of guava juice and jelly.*MS Thesis,* Bangladesh Agriculture University, Mymensingh.

Krochta, J.M. and Mulder-Johnston, C.D., (1997). Edible and biodegradable polymer films: Challenges and Opportunities. *Food Technology,* 51(2): 61-74.

Kumar, S. andBhatnagar, T. (2014). Studies to Enhance the Shelf Life of Fruits Using *Aloe Vera* Based Herbal Coatings: A Review. *Journal of Agriculture and Food Science Technology,* 5(3): 211 218.

Lee, S.K. and Kader, A.A. (2000). Preharvest and postharvest factors influencing vitamin C content of horticultural crops.*Postharvest Biology and Technology,*20: 207–220.

Li, H., and Yu, T. (2000). Effect of chitosan on incidence of brown rot, quality and physiological attributes of postharvest peach fruit. *Journal of the Science of Food and Agriculture,*81: 269-274.

Lima, M.A., Durigan, J.F., de Souza, B.S., andDonadon, J.R. (2003).Post-harvest conservation of guavas using calcium and gibberellic acid together with different plastic films.*RevistaBrasileira de Armazenamento,* 28: 31–40.

Lin, J.Y. and Tang, C.Y. (2007).Determination of total phenolic and flavonoid contents in selected fruits and vegetables, as well as their stimulatory effects on mouse splenocyte proliferation.*Food Chemistry,*101: 140–147.

Lucimara, A., Foratoa, Douglas de Brittob., Jackeline, S. de Rizzoa., Taís A. Gastaldia and Odilio B.G. Assisa.(2015). Effect of cashew gum-carboxymethylcellulose edible coatings in extending the shelf-life of fresh and cut guavas.*Journal of Food Packaging and Shelf Life,*5:68–74.

Martínez-Romero, D., Alburquerque, N., Valverde, J.M., Guillén, F., Castillo, S., Valero,D. and Serrano, M. (2006). Postharvest sweet cherry quality and safety maintenanceby Aloe vera treatment: a new edible coating. *Postharvest Biology and Technology,* 39: 93–100.

MIRANDA, M. *et al.* (2009). Influence of temperature on the drying kinetic, physicochemical properties, and antioxidant capacity of Aloe vera (Aloe barbadensis Miller) gel. *Journal of Food Engineering*, 91(2): 297-304.

Mitra, R.J. and Bose.T.K.(1990). Guava. In: Fruits: Tropical and subtropical. T.K. Bose and S. K. Mitra. Eds. Nayaprokash. Calcatta-6 India.280-303.

*Morton, JF. (1987).*Guava, in Fruits of Warm Climates.*Center for New Crops & Plant Products, Department of Horticulture and Landscape Architecture, Purdue University, West Lafayette, Indiana.*356-363.

Nag, A.R. (1998). Physico-chemical changes of four guava varieties during different stages of ripening. M. S. Thesis, Depertment of Horticulture, BAU, Mymensingh, 75.

Navarro, D., Díaz-Mula, H.M., Guillén, F., Zapata, P.J., Castillo, S., Serrano, M., Valero, D., Martínez-Romero, D. (2011). Reduction of nectarine decay caused by Rhizopusstolonifer, Botrytis cinerea and Penicilliumdigitatum with Aloe vera gel alone or with the addition of thymol.*International Journal of Food Microbiology,* 151: 241–246.

Ni, Y., Turner, D., Yates, K.M. andTizard, I. (2004).Isolation characterization of structural components of Aloevera L. leaf pulp *InternationalImmunopharmacology,*4: 1745-1755.

Nielsen, S. (2003). Food Analysis Laboratory Manual. Kluwer Academic. New York.

Nisperos-Carriedo, M.O., Baldwin, E.A., and Shaw, P.E. (1992).Development of an edible coating for extending postharvest life of selected fruits and vegetables.*Proc. Florida State Horticulture Society*, 104: 122–125.

Ochoa, MR., Kesseler, AG.,Vullioud, MB.and Lozano, JE. (1999). Physical and chemical characteristics of raspberry pulp: storage effect on composition and color. *Lebensmittel-Wissenschaft und Technologie,*32:149–153.

Orwa, C., Mutua, A.,Kindt, R., Jamnadass, R. and Anthony, S. (2009).Agroforestree Database: a tree reference and selection guide version 4.0. *World Agroforestry Centre, Kenya.*

Palaniswmy, K.P. and K.G. Shanmugavelu.(1974). Physico-chemical characters of some guava varieties.*South Indian journal of Horticulture,* 22(1-2): 8-11.

Park, HJ. (1999). Development of advanced edible coatings for fruits.*Trends in Food Science & Technology,* 10(8): 254- 260.

Pearson, D. (1976). Chemical Analysis of Foods. 7th Edn., Church Hill Livingstone, London, UK., 72-73,138-143, 488-496.

Phandis, N.A. (1970). Physico-chemical composition of guava fruits.*Indian Journal of Horticulture*, 27:417-433.

Pierce, RF. (1983). Comparison between the nutritional contents of the aloe gel from conventional and hydroponically grown plants.*Erde international*, 1:37–38.

Pozo, L., Perez Ascorbique I. andVelazquiz, B.(1983). Determination of ascorbic acid in red guava pulp by the 2,6- dichlorophenol indo phenol calorimenteric method with xylem extraction.*Cicucia- Y- Teenica- enla- Agriculture, Citrico-y-others-Frutales*, 6(4): 65-74.

Rahim, N., Gomes, D. J., Watanabe, H., Rahman, S. R., Chomvarin, C., Endtz, H.P., and Alam, M.(2010). Antibacterial activity of Psidiumguajava leaf and bark against multidrug-resistant vibrio cholerae: Implication for cholera control. *Japanese Journal of Infectious Diseases,*63: 271-274.

Rowe, TD.and Park, LM. (1941). Phytochemical study of *Aloe vera*leaf.*Journal of the American Pharmaceutical Association*, 30:262–266.

Schlimme, D.V. and Rooney, M.L. (1994). Packaging of minimally processed fruits and vegetables. In: Wiley RC (ed) Minimally Processed Refrigerated Fruits and Vegetables, Chapman and Hall, 135-182.

Silva, J.M., Correia, L.C.S.A., Moura, N.P., Maciel, M.I.S., andVillar, H.P. (2011).Use of ionising radiation technology as a method of postharvest conservation of guava.*International Journal of Postharvest Technology and Innovation*, 2:168–179.

Singh, S.P., and Pal, R.K. (2008).Controlled atmosphere storage of guava (Psidiumguajava L.) fruit. *Postharvest Biology and Technology*, 47:296–306.

Siqueira, A.M.A., da Costa, J.M.C., Afonso, M.R.A., and Clemente, E. (2011). Pigments of guava paluma cultivar stored under environmental conditions. *African Journal of Food Science*, 5: 320–323.

Siracusa, V., Rocculi, P., Romani, S. and Dalla Rosa, M. (2008). Biodegradable polymers for food packaging: a review. *Trends in Food Science and Technology*, 19: 634.

Smith, S., Geeson, J., and Stow, J. (1987).Production of modified atmospheres in deciduous fruits by the use of films and coatings. *Horticultural Science*, 22(5): 772-776.

Taylor, J.E. (1993). Exotics. In G.B. Seymour, J.E. Taylor, and G.A. Tucker (Eds.), *Biochemistry of fruit ripening*,163–165.

Thaipong, K., Boonprakoba, U., Crosbyb, K., Cisneros-Zevallosc, L. andByrnec, D.H., (2006). Comparison of ABTS, DPPH, FRAP, and ORAC assays for estimating antioxidant activity from guava fruit extracts. *Journal of Food Composition and Analysis, 19*: 669–675.

Tharanathan, R.N. (2003). Biodegradable films and composite coatings: past, present and future. *Trends in Food Science and Technology,* 14: 71.

United States Department Of Agriculture Human Nutrition Information Service., (1982). Agriculture handbook no. 8-9.

Valverde, J.M., Valero, D., Martínez-Romero, D., Guillén, F., Castillo, S. and Serrano, M. (2005). Novel edible coating based on of Aloe vera gel to maintain table grapequality and safety. *Journal of Agricultural and Food Chemistry,* 53: 7807–7813.

Vargas, M., Pastov, C., Chirau, A., Clements, M C., Julian, D. andGunzalez Martinez, C. (2008). Recent advances in edible coatings for fresh and minimally processed fruits.*Critical Reviews in Food Science and Nutrition,*48: 496-511.

Vazquez-Ochoa, R. I., and Colinas-Leon, M. T. (1990).Changes in guavas of three maturity stages in response to temperature and relative humidity.*Horticultural science,* 25: 86–87.

Vila, MTR., Lima, LCO., Boas, VBV, Hojo, ETD., Rodrigues, LJ. and Paula, NRF., (2007). Chemical and biochemical characterization of guavas stored under refrigeration and modified atmosphere. *CiencAgrotec, Lavras*., 31(5): 1435-1442.

Watada, A.E., Ko, N.P. andMinott, D.A. (1996). Factors affecting quality of fresh-cut horticultural products. *Postharvest Biology and Technology*, 9:115-125.

Weinert, I.A.G. and Van Wyk, P.J. (1988). Guava puree with reduced stone cell content: preparation and characteristics of concentrates and nectars. *International Journal of Food Science and Technology*, 23 (5): 501–510.

Xing, Y.G., Li, X.H., Xu, Q.L., Yun, J., Lu, Y.Q. and Tang, Y. (2011). Effects of chitosan coating enriched with cinnamon oil on qualitative properties of sweet pepper (*Capsicum annuum* L.) *Food Chemistry,*124:1443–1450.

Xu, S., Chen, X. and Sun, D.W.(2001).Preservation of kiwifruit coated with an edible film at ambient temperature, *Journal of Food Engineering,* 50: 211-216.

Yaman, O. and Bayoundurh, L. (2002).Effects of an edible coating and cold storage on shelf-life and quality of cherries. *Lebesm-Wiss. U. Technology*, 35:146-150.

Yusof, S., Mohammed, S. and Barak, A. Abu., (1988). Effect of the fruit maturity on the quality and acceptability of guava puree.*Food chemistry,* 30 (10): 45-58.

Zuraidah, N., Justine, Ng Wei Ye and Yusnita, H. (2015). Characteristics of Fresh-Cut Guava Coated with *Aloe vera*Gel as Affected by Different Additives.*Kasetsart Journal of Natural Science,* 49: 111–121.
