# Supplementary material for: Physical and chemical properties of aloe-vera coated guava (Psidium guajava) fruit during refrigerated storage
Source: PLoS One. 2023 Nov 1;18(11):e0293553. doi: 10.1371/journal.pone.0293553 (PMC10619840; doi:10.1371/journal.pone.0293553)
Supplement: Attachment — Submitted filename: Response to reviewers.docx [file pone.0293553.s005.docx]

Dear Reviewers,

According to your suggestion,

1. The title page is arranged according to the rule of the journal where clear title and author lists is updated with their affiliations and indicate the corresponding author with the email address.
2. The line numbers are added in the manuscript.
3. The grammatical errors of the core findings are corrected according to reviewer 02.
4. The grammatical errors in the abstract are corrected according to reviewer 02.
5. The introduction references and grammatical errors are corrected in paragraphs 01, 02, and 03 according to reviewer 02.
6. The 1st paragraph of the material and methods named experimental site is corrected according to the suggestion of reviewer 02.
7. All references to the materials and methods are corrected according to the journal instructions.
8. The guava coating process section is rewritten according to the suggestion of the reviewer.
9. All references in the result and discussion section are rewritten according to the instruction of the journal.
10. The grammatical errors are corrected and the suggestions are maintained according to the suggestion of the reviewer 02.
11. All unnecessary sentences in the paragraphs of the materials and methods section are removed and rewritten according to the suggestion of reviewer 02.
12. The grammatical errors in the conclusion are corrected according to reviewer 02.
13. All references are correctly rewritten according to the instruction of the journal.
