# Supplementary material for: Physical and chemical properties of aloe-vera coated guava (Psidium guajava) fruit during refrigerated storage
Source: PLoS One. 2023 Nov 1;18(11):e0293553. doi: 10.1371/journal.pone.0293553 (PMC10619840; doi:10.1371/journal.pone.0293553)
Supplement: Attachment — Submitted filename: Response to reviewers.docx [file pone.0293553.s006.docx]

Detailed response to reviewers

Manuscript Reference: PONE-D-23-12719R1

Manuscript Title: Physical and chemical properties of Aloe vera coated guava (Psidium guajava) fruit during refrigerated storage.

Author(s): Debashis Kumar Dutta Roy^1^, Md. Asaduzzaman^1,2*^, Tanny Saha^1^, Mst. Nazma Khatun^1,3^

Dear Editor and Reviewers,

Thank you very much for your constructive and useful suggestions. Those comments were valuable and very helpful for revising and improving our paper, as well as for increasing the significance of our research. The responses to editor and reviewer’s comments/suggestions of our manuscript have been answered carefully point-by-point as follows. Some minor mistakes within the manuscript have also been corrected.

Note: All the line numbers in our responses refer to the revised manuscript. The red color in the manuscript indicates the changes made during revision.

**Response to reviewers**

**Reviewer #1:**

Thank you for your positive perspective on the manuscript.

**Reviewer #3:**

Dear Author

According to the evaluation of your research unfortunately it was not contains novelty. And contains many repeated analysis which previously published therefore your work can not consider to publishing in POLS ONE journal.

I mentioned some references as below:

Baldwin EA, Nisperos‐ Carriedo MO, Baker RA. Use of edible coatings to preserve quality of lightly (and slightly) processed products. Critical Reviews in Food Science & Nutrition. 1995 Nov 1;35(6):509-24

Martínez-Romero D, Castillo S, Guillén F, Díaz-Mula HM, Zapata PJ, Valero D, Serrano M. Aloe vera gel coating maintains quality and safety of ready-to-eat pomegranate arils. Postharvest Biology and Technology. 2013 Dec 1;86:107-12

Thanks you for your positive view about the manuscript. This type of work has been previously undertaken elsewhere, but in Bangladesh, it had not occurred before. There may be slight differences between the raw materials and coating materials used here. For Bangladesh, the utilization of fruits and aloe vera in this manner represents a novel and innovative approach.

**Reviewer #4:**

Herein, various concentrations of aloe vera-based coating formulation (25%, 50%, 75%, and 100%) were applied on fresh whole guava by dipping method. A significant effect of aloe vera coating was found over the storage period. Aloe vera treatment lowered the weight loss, and retarded the texture and color compared to the control sample throughout the 28 days of storage. The paper is good and may be published after following revision.

Thank you for your positive view about the manuscript. We have carefully revised the manuscript according to your suggestions. The answer of the individual questions has been written below.

Comments of the answer:

1. Authors already revised the ms. in a good way on the basis of previous comments raised by another reviewer.

Thank you for the compliments.

2. English still needs little bit more polish.

Try my best to polish the manuscript little bit.

3. Abstract needs a little bit more elaboration.

The abstract elaborate again but due to word counts in the editorial dashboard the abstract need to minimize as possible.

4. In keywords give “pH” and remove “Refrigeration”.

The Keyword “Refrigeration” is deleted and “pH” Keyword is added according to the suggestion of the Reviewer 04 in the manuscript and the editorial dashboard.

5. Elaborate introduction section and give some more specific literatures.

The introductions are elaborate and add one more literatures.

6. Is aloe-vera easily available in guava rich countries?

Aloe vera and guava are readily available throughout Bangladesh.

**Reviewer #5:**

The authors tried to answer the reviewers' comments but not satisfactorily. The grammar still needs to be edited. The rule of number and unit(s) needed to be followed. The rule of space after a full stop before a new sentence(s) are put need to be looked into.

Thank you for your positive feedback on the manuscript. We have diligently revised the document based on your valuable suggestions. I apologize for any errors, as English is not my native language, and I sometimes struggle to identify grammatical issues. Nevertheless, I am doing my utmost to ensure the accuracy of the grammar.
