# Supplementary material for: Physical and chemical properties of aloe-vera coated guava (Psidium guajava) fruit during refrigerated storage
Source: PLoS One. 2023 Nov 1;18(11):e0293553. doi: 10.1371/journal.pone.0293553 (PMC10619840; doi:10.1371/journal.pone.0293553)
Supplement: Peer review history

# Peer review: Decision Letter 0

**DOI:** 10.1371/journal.pone.0293553.r001

**Authors:** Awatif Abid Al-Judaibi

5 Jul 2023

PONE-D-23-12719Physical and chemical properties of Aloe vera coated guava (Psidium guajava) fruit during refrigerated storage.PLOS ONE

Dear Dr. Md. Asaduzzaman,

Thank you for submitting your manuscript to PLOS ONE. After careful consideration, we feel that it has merit but does not fully meet PLOS ONE’s publication criteria as it currently stands. Therefore, we invite you to submit a revised version of the manuscript that addresses the points raised during the review process.

Please submit your revised manuscript by Aug 19 2023 11:59PM. If you will need more time than this to complete your revisions, please reply to this message or contact the journal office at plosone@plos.org. When you're ready to submit your revision, log on to https://www.editorialmanager.com/pone/ and select the 'Submissions Needing Revision' folder to locate your manuscript file.

Please include the following items when submitting your revised manuscript:

- A rebuttal letter that responds to each point raised by the academic editor and reviewer(s). You should upload this letter as a separate file labeled 'Response to Reviewers'.
- A marked-up copy of your manuscript that highlights changes made to the original version. You should upload this as a separate file labeled 'Revised Manuscript with Track Changes'.
- An unmarked version of your revised paper without tracked changes. You should upload this as a separate file labeled 'Manuscript'.

If you would like to make changes to your financial disclosure, please include your updated statement in your cover letter. Guidelines for resubmitting your figure files are available below the reviewer comments at the end of this letter.

If applicable, we recommend that you deposit your laboratory protocols in protocols.io to enhance the reproducibility of your results. Protocols.io assigns your protocol its own identifier (DOI) so that it can be cited independently in the future. For instructions see: https://journals.plos.org/plosone/s/submission-guidelines#loc-laboratory-protocols. Additionally, PLOS ONE offers an option for publishing peer-reviewed Lab Protocol articles, which describe protocols hosted on protocols.io. Read more information on sharing protocols at https://plos.org/protocols?utm_medium=editorial-email&utm_source=authorletters&utm_campaign=protocols.

We look forward to receiving your revised manuscript.

Kind regards,

Awatif Abid Al-Judaibi, PhD

Academic Editor

PLOS ONE

Journal Requirements: 

When submitting your revision, we need you to address these additional requirements.

1. Please ensure that your manuscript meets PLOS ONE's style requirements, including those for file naming. The PLOS ONE style templates can be found at

https://journals.plos.org/plosone/s/file?id=wjVg/PLOSOne_formatting_sample_main_body.pdf and

https://journals.plos.org/plosone/s/file?id=ba62/PLOSOne_formatting_sample_title_authors_affiliations.pdf

2. Please note that PLOS ONE has specific guidelines on code sharing for submissions in which author-generated code underpins the findings in the manuscript. In these cases, all author-generated code must be made available without restrictions upon publication of the work. Please review our guidelines at https://journals.plos.org/plosone/s/materials-and-software-sharing#loc-sharing-code and ensure that your code is shared in a way that follows best practice and facilitates reproducibility and reuse.

3. In your Data Availability statement, you have not specified where the minimal data set underlying the results described in your manuscript can be found. PLOS defines a study's minimal data set as the underlying data used to reach the conclusions drawn in the manuscript and any additional data required to replicate the reported study findings in their entirety. All PLOS journals require that the minimal data set be made fully available. For more information about our data policy, please see http://journals.plos.org/plosone/s/data-availability.

Upon re-submitting your revised manuscript, please upload your study’s minimal underlying data set as either Supporting Information files or to a stable, public repository and include the relevant URLs, DOIs, or accession numbers within your revised cover letter. For a list of acceptable repositories, please see http://journals.plos.org/plosone/s/data-availability#loc-recommended-repositories. Any potentially identifying patient information must be fully anonymized.

Important: If there are ethical or legal restrictions to sharing your data publicly, please explain these restrictions in detail. Please see our guidelines for more information on what we consider unacceptable restrictions to publicly sharing data: http://journals.plos.org/plosone/s/data-availability#loc-unacceptable-data-access-restrictions. Note that it is not acceptable for the authors to be the sole named individuals responsible for ensuring data access.

We will update your Data Availability statement to reflect the information you provide in your cover letter.

Additional Editor Comments:

In results section, the subtitle of the result should be written as the subtitle in the materials and methods the Caps letter just in the first letter. Discussion needs to provide some explanation and previous studies' results that agree or disagree with the results of this study.

[Note: HTML markup is below. Please do not edit.]

Reviewers' comments:

Reviewer's Responses to Questions

**Comments to the Author**

1. Is the manuscript technically sound, and do the data support the conclusions?

The manuscript must describe a technically sound piece of scientific research with data that supports the conclusions. Experiments must have been conducted rigorously, with appropriate controls, replication, and sample sizes. The conclusions must be drawn appropriately based on the data presented. 

Reviewer #1: Yes

Reviewer #2: Partly

**********

2. Has the statistical analysis been performed appropriately and rigorously? 

Reviewer #1: Yes

Reviewer #2: N/A

**********

3. Have the authors made all data underlying the findings in their manuscript fully available?

The PLOS Data policy requires authors to make all data underlying the findings described in their manuscript fully available without restriction, with rare exception (please refer to the Data Availability Statement in the manuscript PDF file). The data should be provided as part of the manuscript or its supporting information, or deposited to a public repository. For example, in addition to summary statistics, the data points behind means, medians and variance measures should be available. If there are restrictions on publicly sharing data—e.g. participant privacy or use of data from a third party—those must be specified.

Reviewer #1: Yes

Reviewer #2: No

**********

4. Is the manuscript presented in an intelligible fashion and written in standard English?

PLOS ONE does not copyedit accepted manuscripts, so the language in submitted articles must be clear, correct, and unambiguous. Any typographical or grammatical errors should be corrected at revision, so please note any specific errors here.

Reviewer #1: Yes

Reviewer #2: No

**********

5. Review Comments to the Author

Please use the space provided to explain your answers to the questions above. You may also include additional comments for the author, including concerns about dual publication, research ethics, or publication ethics. (Please upload your review as an attachment if it exceeds 20,000 characters)

Reviewer #1: The literature review in the introduction is well structured and informative providing relevant sources to support the arguments made. The references cited cover a range of topics, including the nutritional value of guava, the mechanisms of post-harvest spoilage, and the benefits of edible coatings, particularly aloe vera.

However, some areas could be improved in the introduction. Firstly, it would be helpful to include a clear statement of the research question or hypothesis to give readers a sense of the main aim of the study. Secondly, some of the information provided could benefit from further clarification, such as the specific causes of post-harvest waste of guava in Bangladesh and the mechanisms by which aloe vera coating improves the shelf life of fruits. Lastly, the introduction would benefit from a stronger concluding paragraph that summarizes the main points and transitions to the methods section of the paper.

Thirdly, the references should be more recent.

The methodology used in this study was appropriate and well-described, making it easy to follow. The authors used analytical grade chemicals and followed standard methods for determining the physical and chemical properties of the coated guava fruits. The results obtained were also presented in a clear and organized manner.

However, there are a few points that need to be addressed by the authors. It would be helpful if they provided more information on the characteristics of the guava fruits used in the study, such as their cultivar, maturity stage, and initial quality. This information would help readers better understand the results obtained and the potential applicability of the Aloe vera gel-based coating to different types of guava fruits.

The paper is well-structured and provides clear information about the methodology used, the results obtained, and the discussion of the findings. The figures and tables used are clear and informative, making it easy for the reader to understand the results. However, the authors did not provide any information about the type of guava used in the study, which can be an important factor in determining the color change during storage.

The paper is generally well-written, but there are some issues with grammar and spelling that need to be addressed. For example, there are some typos in the paper, such as "poly saccharide" instead of "polysaccharide", "discussion" instead of "discussion" Additionally, some of the sentences are too long and complex, which can make it difficult for the reader to follow the argument.

Overall, this study presents an interesting and promising approach to preserving guava using a natural and edible coating material. The authors have provided clear and concise information about the methodology used, the results obtained, and the discussion of the findings. However, some improvements are needed in the writing and editing of the paper. There are also some aspects that could be further elaborated on to strengthen the study. For example, the study could benefit from additional analyses of the microbial and sensory properties of the coated guava, as well as a more detailed discussion of the potential mechanisms behind the observed effects. Additionally, the study could benefit from a larger sample size and a more rigorous statistical analysis to further support the findings. Overall, this study provides a good starting point for future research in this area, but further studies are needed to fully evaluate the potential of aloe vera as a preservation technique for guava.

Reviewer #2: The manuscript is very poorly written. Many sentences need restructuring and clarification of meaning. The figures are not clearly explained. The tables are hard to understand. Overall, the manuscript is not suitable for publication at its current state in this journal.

**********

6. PLOS authors have the option to publish the peer review history of their article (what does this mean?). If published, this will include your full peer review and any attached files.

If you choose “no”, your identity will remain anonymous but your review may still be made public.

**Do you want your identity to be public for this peer review?** For information about this choice, including consent withdrawal, please see our Privacy Policy.

Reviewer #1: **Yes: **Alao Jude Oluwapelumi

Reviewer #2: No

**********

[NOTE: If reviewer comments were submitted as an attachment file, they will be attached to this email and accessible via the submission site. Please log into your account, locate the manuscript record, and check for the action link "View Attachments". If this link does not appear, there are no attachment files.]

While revising your submission, please upload your figure files to the Preflight Analysis and Conversion Engine (PACE) digital diagnostic tool, https://pacev2.apexcovantage.com/. PACE helps ensure that figures meet PLOS requirements. To use PACE, you must first register as a user. Registration is free. Then, login and navigate to the UPLOAD tab, where you will find detailed instructions on how to use the tool. If you encounter any issues or have any questions when using PACE, please email PLOS at figures@plos.org. Please note that Supporting Information files do not need this step.

Attachment Submitted filename: Reviewed_PONE-D-23-12719.docx Click here for additional data file.

# Author comment: Author response to Decision Letter 0

**DOI:** 10.1371/journal.pone.0293553.r002

13 Aug 2023

Dear Reviewer,

It gives me great pleasure to work under your supervision. Your excellent recommendations have helped me to improve my manuscript, and I have learned a lot of new things which have been of immense assistance to me. I have made every effort to follow your recommendations. I kindly request your further guidance by providing additional information.

The changes are 

1. The title page is arranged according to the rule of the journal where clear title and author lists is updated with their affiliations and indicate the corresponding author with the email address.

2. The line numbers are added in the manuscript.

3. The grammatical errors of the core findings are corrected according to reviewer 02.

4. The grammatical errors in the abstract are corrected according to reviewer 02.

5. The introduction references and grammatical errors are corrected in paragraphs 01, 02, and 03 according to reviewer 02.

6. The 1st paragraph of the material and methods named experimental site is corrected according to the suggestion of reviewer 02.

7. All references to the materials and methods are corrected according to the journal instructions.

8. The guava coating process section is rewritten according to the suggestion of the reviewer.

9. All references in the result and discussion section are rewritten according to the instruction of the journal.

10. The grammatical errors are corrected and the suggestions are maintained according to the suggestion of the reviewer 02.

11. All unnecessary sentences in the paragraphs of the materials and methods section are removed and rewritten according to the suggestion of reviewer 02.

12. The grammatical errors in the conclusion are corrected according to reviewer 02.

13. All references are correctly rewritten according to the instruction of the journal.

Thank you.

Yours sincerely,

Md. Asaduzzaman

Attachment Submitted filename: Response to reviewers.docx Click here for additional data file.

# Peer review: Decision Letter 1

**DOI:** 10.1371/journal.pone.0293553.r003

**Authors:** Awatif Abid Al-Judaibi

11 Sep 2023

PONE-D-23-12719R1Physical and chemical properties of Aloe vera coated guava (Psidium guajava) fruit during refrigerated storage.PLOS ONE

Dear Dr. Asaduzzaman,

Thank you for submitting your manuscript to PLOS ONE. After careful consideration, we feel that it has merit but does not fully meet PLOS ONE’s publication criteria as it currently stands. Therefore, we invite you to submit a revised version of the manuscript that addresses the points raised during the review process.

Please submit your revised manuscript by Oct 26 2023 11:59PM. If you will need more time than this to complete your revisions, please reply to this message or contact the journal office at plosone@plos.org. When you're ready to submit your revision, log on to https://www.editorialmanager.com/pone/ and select the 'Submissions Needing Revision' folder to locate your manuscript file.

Please include the following items when submitting your revised manuscript:

- A rebuttal letter that responds to each point raised by the academic editor and reviewer(s). You should upload this letter as a separate file labeled 'Response to Reviewers'.
- A marked-up copy of your manuscript that highlights changes made to the original version. You should upload this as a separate file labeled 'Revised Manuscript with Track Changes'.
- An unmarked version of your revised paper without tracked changes. You should upload this as a separate file labeled 'Manuscript'.

If you would like to make changes to your financial disclosure, please include your updated statement in your cover letter. Guidelines for resubmitting your figure files are available below the reviewer comments at the end of this letter.

If applicable, we recommend that you deposit your laboratory protocols in protocols.io to enhance the reproducibility of your results. Protocols.io assigns your protocol its own identifier (DOI) so that it can be cited independently in the future. For instructions see: https://journals.plos.org/plosone/s/submission-guidelines#loc-laboratory-protocols. Additionally, PLOS ONE offers an option for publishing peer-reviewed Lab Protocol articles, which describe protocols hosted on protocols.io. Read more information on sharing protocols at https://plos.org/protocols?utm_medium=editorial-email&utm_source=authorletters&utm_campaign=protocols.

We look forward to receiving your revised manuscript.

Kind regards,

Awatif Abid Al-Judaibi, PhD

Academic Editor

PLOS ONE

Journal Requirements:

Please review your reference list to ensure that it is complete and correct. If you have cited papers that have been retracted, please include the rationale for doing so in the manuscript text, or remove these references and replace them with relevant current references. Any changes to the reference list should be mentioned in the rebuttal letter that accompanies your revised manuscript. If you need to cite a retracted article, indicate the article’s retracted status in the References list and also include a citation and full reference for the retraction notice.

[Note: HTML markup is below. Please do not edit.]

Reviewers' comments:

Reviewer's Responses to Questions

**Comments to the Author**

1. If the authors have adequately addressed your comments raised in a previous round of review and you feel that this manuscript is now acceptable for publication, you may indicate that here to bypass the “Comments to the Author” section, enter your conflict of interest statement in the “Confidential to Editor” section, and submit your "Accept" recommendation.

Reviewer #1: All comments have been addressed

Reviewer #3: All comments have been addressed

Reviewer #4: All comments have been addressed

Reviewer #5: (No Response)

**********

2. Is the manuscript technically sound, and do the data support the conclusions?

The manuscript must describe a technically sound piece of scientific research with data that supports the conclusions. Experiments must have been conducted rigorously, with appropriate controls, replication, and sample sizes. The conclusions must be drawn appropriately based on the data presented. 

Reviewer #1: Yes

Reviewer #3: No

Reviewer #4: Yes

Reviewer #5: Partly

**********

3. Has the statistical analysis been performed appropriately and rigorously? 

Reviewer #1: N/A

Reviewer #3: Yes

Reviewer #4: Yes

Reviewer #5: Yes

**********

4. Have the authors made all data underlying the findings in their manuscript fully available?

The PLOS Data policy requires authors to make all data underlying the findings described in their manuscript fully available without restriction, with rare exception (please refer to the Data Availability Statement in the manuscript PDF file). The data should be provided as part of the manuscript or its supporting information, or deposited to a public repository. For example, in addition to summary statistics, the data points behind means, medians and variance measures should be available. If there are restrictions on publicly sharing data—e.g. participant privacy or use of data from a third party—those must be specified.

Reviewer #1: Yes

Reviewer #3: Yes

Reviewer #4: Yes

Reviewer #5: Yes

**********

5. Is the manuscript presented in an intelligible fashion and written in standard English?

PLOS ONE does not copyedit accepted manuscripts, so the language in submitted articles must be clear, correct, and unambiguous. Any typographical or grammatical errors should be corrected at revision, so please note any specific errors here.

Reviewer #1: Yes

Reviewer #3: Yes

Reviewer #4: Yes

Reviewer #5: No

**********

6. Review Comments to the Author

Please use the space provided to explain your answers to the questions above. You may also include additional comments for the author, including concerns about dual publication, research ethics, or publication ethics. (Please upload your review as an attachment if it exceeds 20,000 characters)

Reviewer #1: (No Response)

Reviewer #3: Dear Author

According to the evaluation of your research unfortunately it was not contains novelty . and contains many repeated analysis which previously published therefore your work can not consider to publishing in POLS ONE journal

I mentioned some references as below:

Baldwin EA, Nisperos‐ Carriedo MO, Baker RA. Use of edible coatings to preserve

quality of lightly (and slightly) processed products. Critical Reviews in Food Science &

Nutrition. 1995 Nov 1;35(6):509-24

Martínez-Romero D, Castillo S, Guillén F, Díaz-Mula HM, Zapata PJ, Valero D,

Serrano M. Aloe vera gel coating maintains quality and safety of ready-to-eat

pomegranate arils. Postharvest Biology and Technology. 2013 Dec 1;86:107-12

Reviewer #4: Herein, various concentrations of aloe vera-based coating formulation (25%, 50%, 75%, and 100%) were applied on fresh whole guava by dipping method. A significant effect of aloe vera coating was found over the storage period. Aloe vera treatment lowered the weight loss, and retarded the texture and color compared to the control sample throughout the 28 days of storage. The paper is good and may be published after following revision.

1. Authors already revised the ms. in a good way on the basis of previous comments raised by another reviewer.

2. English still needs little bit more polish.

3. Abstract needs a little bit more elaboration.

4. In keywords give “pH” and remove “Refrigeration”.

5. Elaborate introduction section and give some more specific literatures.

6. Is aloe-vera easily available in guava rich countries?

Reviewer #5: The authors tried to answer the reviewers' comments but not satisfactorily. The grammar still needs to be edited. The rule of number and unit(s) needed to be followed. The rule of space after a full stop before a new sentence(s) are put need to be looked into.

**********

7. PLOS authors have the option to publish the peer review history of their article (what does this mean?). If published, this will include your full peer review and any attached files.

If you choose “no”, your identity will remain anonymous but your review may still be made public.

**Do you want your identity to be public for this peer review?** For information about this choice, including consent withdrawal, please see our Privacy Policy.

Reviewer #1: **Yes: **Alao Jude Oluwapelumi

Reviewer #3: No

Reviewer #4: No

Reviewer #5: **Yes: **Prof. Modinah A. O. Abdul Raheem

**********

[NOTE: If reviewer comments were submitted as an attachment file, they will be attached to this email and accessible via the submission site. Please log into your account, locate the manuscript record, and check for the action link "View Attachments". If this link does not appear, there are no attachment files.]

While revising your submission, please upload your figure files to the Preflight Analysis and Conversion Engine (PACE) digital diagnostic tool, https://pacev2.apexcovantage.com/. PACE helps ensure that figures meet PLOS requirements. To use PACE, you must first register as a user. Registration is free. Then, login and navigate to the UPLOAD tab, where you will find detailed instructions on how to use the tool. If you encounter any issues or have any questions when using PACE, please email PLOS at figures@plos.org. Please note that Supporting Information files do not need this step.

# Author comment: Author response to Decision Letter 1

**DOI:** 10.1371/journal.pone.0293553.r004

20 Sep 2023

Response to reviewers

Reviewer #1:

Thank you for your positive perspective on the manuscript.

Reviewer #3:

Dear Author

According to the evaluation of your research unfortunately it was not contains novelty. And contains many repeated analysis which previously published therefore your work can not consider to publishing in POLS ONE journal.

I mentioned some references as below:

Baldwin EA, Nisperos‐ Carriedo MO, Baker RA. Use of edible coatings to preserve quality of lightly (and slightly) processed products. Critical Reviews in Food Science & Nutrition. 1995 Nov 1;35(6):509-24

Martínez-Romero D, Castillo S, Guillén F, Díaz-Mula HM, Zapata PJ, Valero D, Serrano M. Aloe vera gel coating maintains quality and safety of ready-to-eat pomegranate arils. Postharvest Biology and Technology. 2013 Dec 1;86:107-12

Thanks you for your positive view about the manuscript. This type of work has been previously undertaken elsewhere, but in Bangladesh, it had not occurred before. There may be slight differences between the raw materials and coating materials used here. For Bangladesh, the utilization of fruits and aloe vera in this manner represents a novel and innovative approach.

Reviewer #4:

Herein, various concentrations of aloe vera-based coating formulation (25%, 50%, 75%, and 100%) were applied on fresh whole guava by dipping method. A significant effect of aloe vera coating was found over the storage period. Aloe vera treatment lowered the weight loss, and retarded the texture and color compared to the control sample throughout the 28 days of storage. The paper is good and may be published after following revision.

Thank you for your positive view about the manuscript. We have carefully revised the manuscript according to your suggestions. The answer of the individual questions has been written below.

Comments of the answer:

1. Authors already revised the ms. in a good way on the basis of previous comments raised by another reviewer.

Thank you for the compliments.

2. English still needs little bit more polish.

Try my best to polish the manuscript little bit.

3. Abstract needs a little bit more elaboration.

The abstract elaborate again but due to word counts in the editorial dashboard the abstract need to minimize as possible.

4. In keywords give “pH” and remove “Refrigeration”.

The Keyword “Refrigeration” is deleted and “pH” Keyword is added according to the suggestion of the Reviewer 04 in the manuscript and the editorial dashboard.

5. Elaborate introduction section and give some more specific literatures.

The introductions are elaborate and add one more literatures.

6. Is aloe-vera easily available in guava rich countries?

Aloe vera and guava are readily available throughout Bangladesh.

Reviewer #5:

The authors tried to answer the reviewers' comments but not satisfactorily. The grammar still needs to be edited. The rule of number and unit(s) needed to be followed. The rule of space after a full stop before a new sentence(s) are put need to be looked into.

Thank you for your positive feedback on the manuscript. We have diligently revised the document based on your valuable suggestions. I apologize for any errors, as English is not my native language, and I sometimes struggle to identify grammatical issues. Nevertheless, I am doing my utmost to ensure the accuracy of the grammar.

Attachment Submitted filename: Response to reviewers.docx Click here for additional data file.

# Peer review: Decision Letter 2

**DOI:** 10.1371/journal.pone.0293553.r005

**Authors:** Awatif Abid Al-Judaibi

16 Oct 2023

Physical and chemical properties of Aloe vera coated guava (Psidium guajava) fruit during refrigerated storage.

PONE-D-23-12719R2

Dear Dr. Md. Asaduzzaman,

We’re pleased to inform you that your manuscript has been judged scientifically suitable for publication and will be formally accepted for publication once it meets all outstanding technical requirements.

Within one week, you’ll receive an e-mail detailing the required amendments. When these have been addressed, you’ll receive a formal acceptance letter and your manuscript will be scheduled for publication.

An invoice for payment will follow shortly after the formal acceptance. To ensure an efficient process, please log into Editorial Manager at http://www.editorialmanager.com/pone/, click the 'Update My Information' link at the top of the page, and double check that your user information is up-to-date. If you have any billing related questions, please contact our Author Billing department directly at authorbilling@plos.org.

If your institution or institutions have a press office, please notify them about your upcoming paper to help maximize its impact. If they’ll be preparing press materials, please inform our press team as soon as possible -- no later than 48 hours after receiving the formal acceptance. Your manuscript will remain under strict press embargo until 2 pm Eastern Time on the date of publication. For more information, please contact onepress@plos.org.

Kind regards,

Awatif Abid Al-Judaibi, PhD

Academic Editor

PLOS ONE

Additional Editor Comments (optional):

Dear authors,

Thank you for your response, and according to reviewer comments, kindly, be sure to revise the manuscript according to reviewer's comments.

Reviewers' comments:

Reviewer's Responses to Questions

**Comments to the Author**

1. If the authors have adequately addressed your comments raised in a previous round of review and you feel that this manuscript is now acceptable for publication, you may indicate that here to bypass the “Comments to the Author” section, enter your conflict of interest statement in the “Confidential to Editor” section, and submit your "Accept" recommendation.

Reviewer #4: All comments have been addressed

Reviewer #5: (No Response)

**********

2. Is the manuscript technically sound, and do the data support the conclusions?

The manuscript must describe a technically sound piece of scientific research with data that supports the conclusions. Experiments must have been conducted rigorously, with appropriate controls, replication, and sample sizes. The conclusions must be drawn appropriately based on the data presented. 

Reviewer #4: Yes

Reviewer #5: Yes

**********

3. Has the statistical analysis been performed appropriately and rigorously? 

Reviewer #4: I Don't Know

Reviewer #5: Yes

**********

4. Have the authors made all data underlying the findings in their manuscript fully available?

The PLOS Data policy requires authors to make all data underlying the findings described in their manuscript fully available without restriction, with rare exception (please refer to the Data Availability Statement in the manuscript PDF file). The data should be provided as part of the manuscript or its supporting information, or deposited to a public repository. For example, in addition to summary statistics, the data points behind means, medians and variance measures should be available. If there are restrictions on publicly sharing data—e.g. participant privacy or use of data from a third party—those must be specified.

Reviewer #4: Yes

Reviewer #5: Yes

**********

5. Is the manuscript presented in an intelligible fashion and written in standard English?

PLOS ONE does not copyedit accepted manuscripts, so the language in submitted articles must be clear, correct, and unambiguous. Any typographical or grammatical errors should be corrected at revision, so please note any specific errors here.

Reviewer #4: Yes

Reviewer #5: No

**********

6. Review Comments to the Author

Please use the space provided to explain your answers to the questions above. You may also include additional comments for the author, including concerns about dual publication, research ethics, or publication ethics. (Please upload your review as an attachment if it exceeds 20,000 characters)

Reviewer #4: Author have resolved all comments raised by reviewer point by point. Now ready for accept in current form.

Reviewer #5: The second sentence of the abstract need to be rephrased as two sentences, other corrections on the abstract are noted in the manuscript.

The author should take cognizant of the reference especially et al.,.

The rule of number and unit of a space in between should be followed.

Table and figure name should be at the top of the table. The numbering of the table and figure should be 1.0 instead of 01

The author should implore the service of English expert to improve the communication language of the journal

**********

7. PLOS authors have the option to publish the peer review history of their article (what does this mean?). If published, this will include your full peer review and any attached files.

If you choose “no”, your identity will remain anonymous but your review may still be made public.

**Do you want your identity to be public for this peer review?** For information about this choice, including consent withdrawal, please see our Privacy Policy.

Reviewer #4: No

Reviewer #5: **Yes: **Modinah Adenike Oladayo ABDUL RAHEEM

**********

Attachment Submitted filename: PONE-D-23-12719_reviewer.pdf-11.pdf Click here for additional data file.

# Editor report: Acceptance letter

**DOI:** 10.1371/journal.pone.0293553.r006

**Authors:** Awatif Abid Al-Judaibi

23 Oct 2023

PONE-D-23-12719R2 

Physical and chemical properties of Aloe-vera coated guava (Psidium guajava) fruit during refrigerated storage. 

Dear Dr. Asaduzzaman:

I'm pleased to inform you that your manuscript has been deemed suitable for publication in PLOS ONE. Congratulations! Your manuscript is now with our production department. 

If your institution or institutions have a press office, please let them know about your upcoming paper now to help maximize its impact. If they'll be preparing press materials, please inform our press team within the next 48 hours. Your manuscript will remain under strict press embargo until 2 pm Eastern Time on the date of publication. For more information please contact onepress@plos.org.

If we can help with anything else, please email us at plosone@plos.org. 

Thank you for submitting your work to PLOS ONE and supporting open access. 

Kind regards, 

PLOS ONE Editorial Office Staff

on behalf of

Professor Awatif Abid Al-Judaibi 

Academic Editor

PLOS ONE
